# The application of nanoparticles-based ferroptosis, pyroptosis and autophagy in cancer immunotherapy

**DOI:** 10.1186/s12951-024-02297-8

**Published:** 2024-03-07

**Authors:** Wen Deng, Haojie Shang, Yonghua Tong, Xiao Liu, Qiu Huang, Yu He, Jian Wu, Xiaozhuo Ba, Zhiqiang Chen, Yuan Chen, Kun Tang

**Affiliations:** 1 Department of Urology, Tongji Hospital, Tongji Medical College, Huazhong University of Science and Technology, Wuhan, 430030 China; 2 Department of Geriatric Medicine, Tongji Hospital, Tongji Medical College, Huazhong University of Science and Technology, Wuhan, 430030 China

**Keywords:** Ferroptosis, Pyroptosis, Autophagy, Nanomaterials, Cancer immunotherapy

## Abstract

Immune checkpoint blockers (ICBs) have been applied for cancer therapy and achieved great success in the field of cancer immunotherapy. Nevertheless, the broad application of ICBs is limited by the low response rate. To address this issue, increasing studies have found that the induction of immunogenic cell death (ICD) in tumor cells is becoming an emerging therapeutic strategy in cancer treatment, not only straightly killing tumor cells but also enhancing dying cells immunogenicity and activating antitumor immunity. ICD is a generic term representing different cell death modes containing ferroptosis, pyroptosis, autophagy and apoptosis. Traditional chemotherapeutic agents usually inhibit tumor growth based on the apoptotic ICD, but most tumor cells are resistant to the apoptosis. Thus, the induction of non-apoptotic ICD is considered to be a more efficient approach for cancer therapy. In addition, due to the ineffective localization of ICD inducers, various types of nanomaterials have been being developed to achieve targeted delivery of therapeutic agents and improved immunotherapeutic efficiency. In this review, we briefly outline molecular mechanisms of ferroptosis, pyroptosis and autophagy, as well as their reciprocal interactions with antitumor immunity, and then summarize the current progress of ICD-induced nanoparticles based on different strategies and illustrate their applications in the cancer therapy.

**Graphical Abstract:**

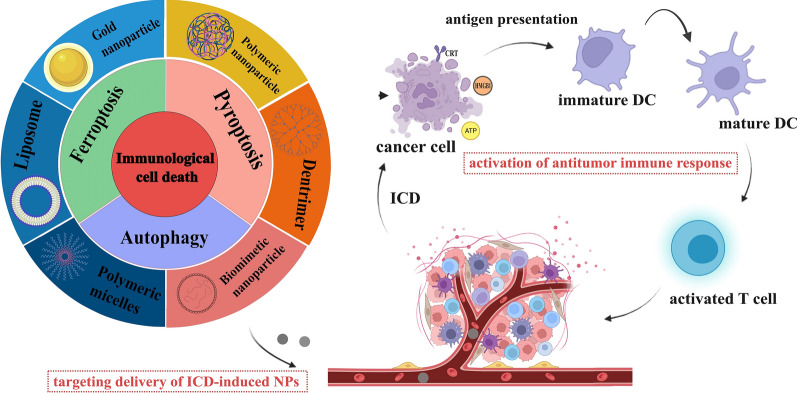

## Background

Cancer immunotherapy, an innovative approach in the field of tumors treatment today, offers several advantages over conventional antitumor therapy in terms of prolonging both progression-free survival and overall survival [1, 2]. While conventional therapies primarily target the tumor itself, the tumor microenvironment (TME) is a rich and complex milieu. In cases where specific treatment strategy exclusively focuses on the tumor without addressing the TME, the desired effects are sometimes elusive. In contrast, immunotherapy is designed to target the tumor microenvironment, effectively reversing immunosuppression and restoring the immune system's ability to attack tumor cells. In this section, we would like to provide a brief overview of various therapy approaches used in tumor immunotherapy, containing of molecular targeted therapy, adoptive cell therapy, cytokine therapy, and neoplastic vaccine immunotherapy, immune checkpoint inhibitors, each with distinct mechanisms of action.

**Fig. 1:**
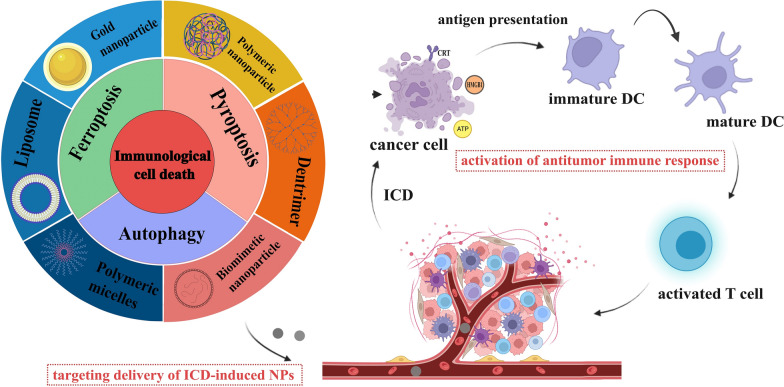
Graphical abstract

Molecular targeted therapy represents an innovative treatment approach that focuses on key factors associated with the development of tumor cells. These factors include cell signaling pathways, cytokine receptors, anti-tumor angiogenesis, proto-oncogenes, oncogenes, and more. The objective of this therapy is to counteract these malignancy-promoting behaviors at the molecular level. In addition, it offers enhanced precision and selectivity at the molecular and cellular levels, enabling the efficient and selective eradication of tumor cells while minimizing harm to healthy tissues. Monoclonal antibodies play a prominent role in biologically targeted therapies. A monoclonal antibody (mAb) is a specialized antibody that specifically targets a single epitope region on an antigen and has become an integral part of clinical practice for inhibiting and sometimes eradicating tumors, typically produced using hybridoma technology [3, 4]. It achieves this by recruiting T cells to the tumor site, directly targeting tumor cells and binding to antigens on the tumor's surface. Monoclonal antibodies are primarily used in the treatment of breast cancer, colon cancer, and lymphoma, among others [5, 6]. Notably, approximately 33 cancer therapeutic antibodies have stepped into an advanced stage of clinical research for various types of cancers, such as chronic lymphocytic leukemia, multiple myeloma, breast cancer, and bladder cancer [7]. The global monoclonal antibody market is currently on a promising trajectory, and with ongoing enhancement and development, monoclonal antibody therapy is poised to become the preferred weapon of choice in cancer treatment (Fig. 1).

Adoptive immune cell therapy (ACT) is an active and promising area in tumor biotherapy. It involves transferring immune cells, both specific and non-specific, with anti-tumor properties to patients with tumors. These transferred cells can directly kill tumor cells or stimulate the body's immune response to target and eliminate the tumor cells [8]. ACT can be categorized into several types, including Engineered T-cell receptor (TCR) therapy, Chimeric antigen receptor (CAR) T-cell therapy, Natural killer (NK) cell therapy, Tumor-infiltrating lymphocyte (TIL) therapy. Among these, CAR-T has been particularly successful, especially in treating B-cell lymphoma, and has received approval for clinical use due to its demonstrated effectiveness [9]. It's essential to note that ACT therapy can potentially lead to severe adverse effects, and while most acute symptoms can be treated if detected promptly, it highlights the need for standardized guidelines to recognize and manage ACT-induced damages [10]. The development of standardized guidelines will ensure the safe and effective application of this promising immunotherapeutic approach.

Cytokines are instrumental in tumor pathogenesis. These signaling molecules, which are released during infection, inflammation, and immune responses, can have both inhibitory and promoting effects on tumor development. Cytokines influence cell growth, apoptosis regulation, and tumor cell metastasis, making them crucial factors in the context of cancer. In-depth studies of cytokine interactions between tumor cells and normal cells provide valuable insights for improving tumor immunotherapy [11]. Commonly used cytokines in anti-tumor immunotherapy include IL-2, IL-12, INF-γ, and TNF. They exert their tumor-killing effects through several mechanisms: ① Up-regulating the expression and secretion of surface molecules and receptors on immune cells; ② Enhancing the immune surveillance function of the body, including promoting the proliferation and differentiation of T cells, maturation of cytotoxic T lymphocytes (CTLs), stimulation of antibody production by B cells, and increasing the activity of natural killer (NK) cells and other anti-tumor immune responses; ③ Promoting the release of lymphotoxin and effector molecules from immune cells to kill tumors; ④ Encouraging the expression of major histocompatibility complex (MHC) molecules on tumor cells, thus enhancing the immunogenicity of tumor cells and their sensitivity to immune effector cells; ⑤ Certain cytokines can directly induce apoptosis in tumor cells, such as TNF. These mechanisms collectively illustrate the multifaceted roles that cytokines play in the regulation of immune responses and their potential to enhance tumor immunotherapy [12, 13].

Cancer vaccines are among the most eagerly anticipated developments in medical science for the general public. While traditional vaccines are designed to prevent a wide range of infectious diseases, cancer vaccines, which have gained significant attention in recent years, have a different goal: to harness the body's immune system to target and eliminate tumor cells. Cancer vaccine therapy has demonstrated the ability to induce regression of large tumors throughout the body and extend the survival of cancer patients [14]. Compared with other immunotherapies, cancer vaccines offer unique advantages. These advantages include the ability to target intracellular antigens in addition to tumor-specific surface antigens and even the potential to trigger entirely new tumor-specific T-cell responses. The fundamental mechanism of cancer vaccines involves the uptake of tumor antigens by antigen-presenting cells (APCs), which then present these antigens on HLA-I molecules to CD8 + T cells, resulting in stimulating an immune response against these tumor-specific antigens [15]. The first and currently only cancer vaccine in the world to receive approval from the U.S. Food and Drug Administration is Provenge (sipuleucel-T). Additionally, dendritic cell (DC) vaccines have made significant breakthroughs in numerous clinical trials [16]. However, the number of clinical trials involving cancer vaccines is relatively limited, and researchers are working to further explore and confirm their therapeutic efficacy, as well as establish well-defined principles for their use. The potential of cancer vaccines to revolutionize cancer treatment is a subject of ongoing research and development [17].

Recently, immunotherapy based on ICBs has made significant progress in tumor therapy. Immune checkpoints are mechanisms within the body that cancer cells can exploit to evade the immune system. Immune checkpoint molecules, found on immune cells, serve as regulators in the immune system, mainly playing an inhibitory role. These molecules are crucial for maintaining self-tolerance, preventing autoimmune reactions, and controlling the timing and intensity of immune responses to minimize tissue damage. However, cancer cells can hijack these mechanisms to inhibit the immune response, preventing the body from mounting an effective anti-tumor immune reaction. Notable tumor-related immune checkpoint molecules include PD1, CTLA4, Tim3, and LAG3, with PD1 and CTLA4 being the most extensively studied [18]. ICBs are designed to target these specific immune checkpoints. Their primary function is to block the interaction between tumor cells expressing immune checkpoints and immune cells, thus counteracting the inhibitory effect of tumor cells on the immune system. Immune checkpoint therapy has shown promise by providing long-lasting clinical responses and improving overall survival [19].

Challenges in this field mainly include addressing drug resistance and reducing the incidence of immune-related adverse events. In the future, immune checkpoint therapies are expected to expand into various areas of oncology and be developed as part of combination treatments, including surgery, radiation, chemotherapy, targeted therapies, and other immunotherapies. The future of immune checkpoint therapy holds significant promise, offering opportunities to enhance the prognosis for cancer patients. Nevertheless, in the present, only a minority of patients with a majority of tumor types are sensitive to ICBs, partly because of the lack of tumor-infiltrating immune cells, which severely restricts the utilization of ICBs. The conversion of the immunosilent tumors into immunostimulatory tumors that can respond to ICBs remains a difficult problem in the field of cancer immunotherapy [20].

To improve the effect of ICBs in tumor immunotherapy, induction of ICD, an emerging cancer therapy strategy, has been used to treat many types of tumors. ICD is a collective name [21−24]. Specifically, ferroptosis, pyroptosis and autophagy show a synergistic antitumor immune response. ICD features the release or secret of discrete signals a.k.a. danger-associated molecular patterns (DAMPs) by dying or injured cells, which then trigger cellular immunity as crucial adjuvants [25, 26]. DAMPs can be recognized by phagocytosis receptors to trigger the antigen presentation of antigen-presenting cells (APCs), which ultimately activates CD8^+^ T cells against the cancer cells [27, 28]. The entire process further indicates that ICD acts as a positive role in tumor immunotherapy. Overall, DAMPs binding to receptors on immune cells can initiate several cellular cascades, activating geneogenous and adaptive immune responses [29]. Therefore, targeted tumor therapies based on ferroptosis, pyroptosis and autophagy may exert potent antitumor activity when combined with immunotherapy, even in ICBs-resistant tumors (Tables 1, 2, 3).

**Table 1:**
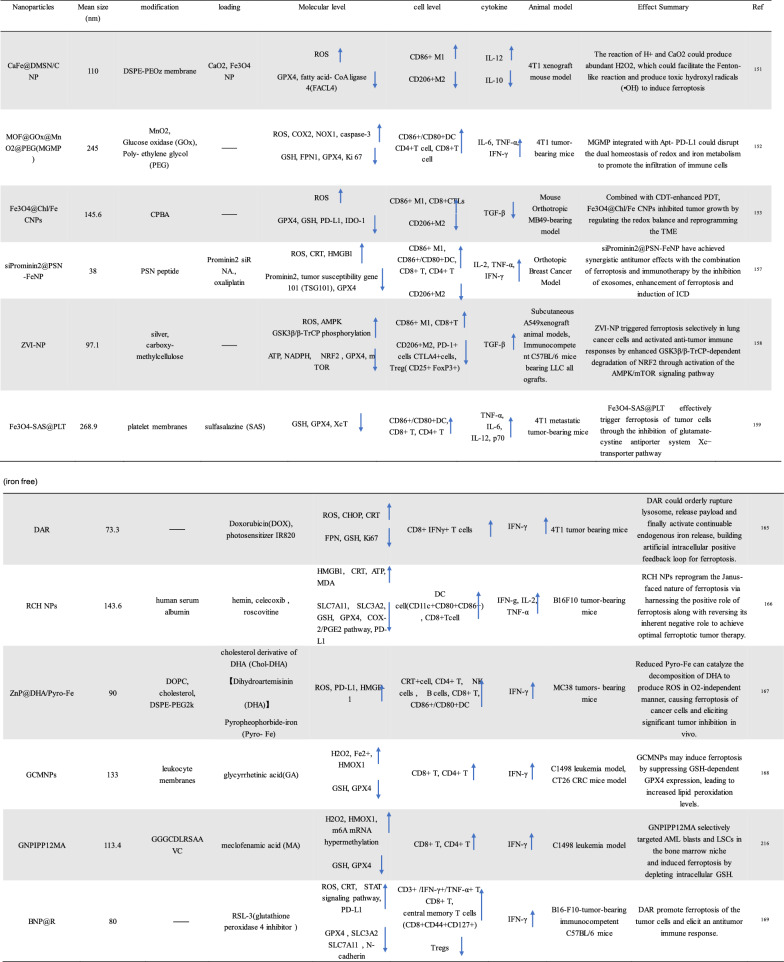
Summary of ferroptosis-induced nanoparticles (iron base)

↓Reduced level; ↑Elevated level

**Table 2:**
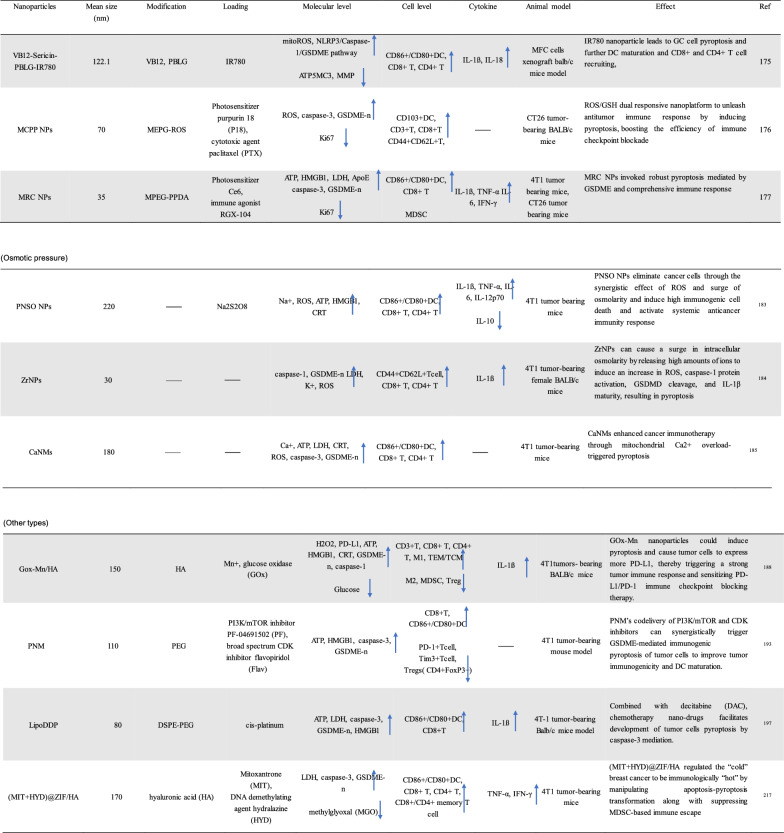
Summary of pyroptosis-induced nanoparticles (PDT/PTT)

↓Reduced level; ↑Elevated level

**Table 3:**
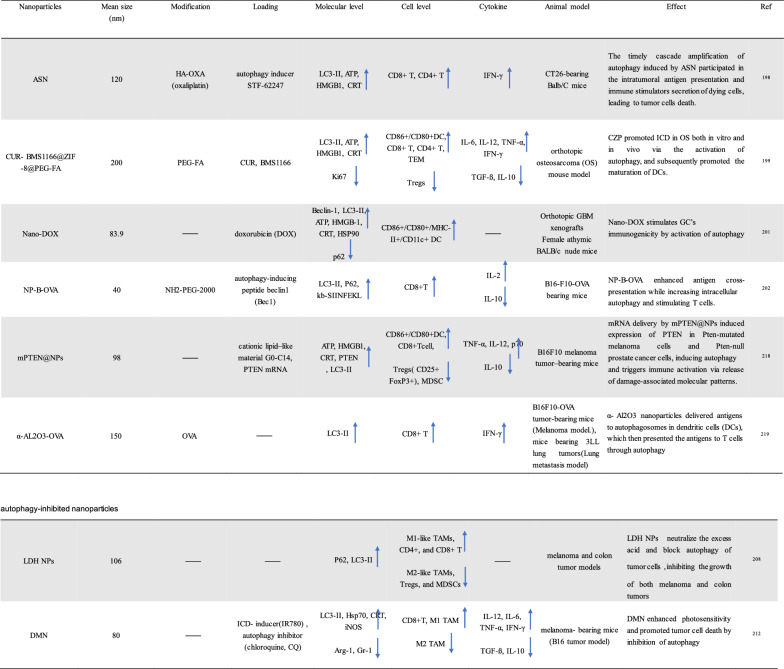
Summary of autophagy-induced nanoparticles

↓Reduced level; ↑Elevated level

Activated by chemotherapy, radiation, or other physical cues, ICD can further induce the immune response, but the use of conventional ICD inducers is restricted by challenges of uncertainty about their security and efficacy [30]. Besides, the number of antitumor therapies capable of activating ICD in experimental tumor therapeutics is also limited and even much less in clinic [31]. To eliminate these limitations, nanomaterials-mediated antitumor therapies have been applied to enhance the immunogenicity of dying tumor cells [30]. Specifically, nanoparticles are able to prevent the therapeutic agents from fast clearance and provide effective, targeted delivery of immunomodulatory agents towards the tumor cells along with their controlled release and the attenuated toxicity, increasing the antitumor immune-efficiency, promoting the curative effect and decreasing adverse reactions [32, 33].

The application of a wide range of nanoparticles can overcome essential difficulties and requirements in regulating death modalities of tumor cells and their immunogenicity [34, 35]. For example, as a new antitumor treatment approach, phototherapeutic agents are usually employed to selectively ablate cancer cells under near-infrared light irradiation, when these physical therapies are combined with nanoparticle delivery systems, their range, duration, and efficacy could be accurately modulated by altering the site, time, and power of irradiation [26].

In the review, we firstly elaborate the features and molecular mechanisms of ICD, including ferroptosis, pyroptosis and autophagy, as well as their correlations with antitumor immunity. We also introduce different strategies of ICD induction mediated by nanoparticles and further illustrate the applications of ICD-induced nanoparticles in tumor immunotherapy. At last, we propose a short discussion and expectation about future challenges, perspectives and opportunities.

## Ferroptosis, pyroptosis, autophagy and antitumor immunity

### Ferroptosis in antitumor immunity

Ferroptosis is a recently discovered immunogenic cell death modality, which is different from apoptosis and characterized by the lethal accumulation of iron-dependent lipid peroxide. As cancer cells are usually resistant to apoptosis inherently, ferroptosis has attracted much attention for its efficacy in suppressing tumor progress [36].

The canonical pathway of ferroptosis involves the dysfunction of cytoprotective mechanisms against lipid ROS damage and requires excess cellular polyunsaturated fatty acids (PUFAs). Iron-catalyzed inordinate peroxidation of PUFAs is the core characteristic in ferroptosis process. The continuous consumption of PUFAs in the plasma membrane changes the fluidity and structure of the membrane, aggrandizing permeability and spoiling membrane integrity [37, 38]. Other subcellular locations can also suffer damages resulting from lipid peroxidation. One of the most typical is the mitochondrion, which can be subjected to severe stress leading to dysfunction and cytological alterations, such as shrinkage and reduction of mitochondrial cristae [37, 39, 40]. In addition, ferroptosis sensitivity is dependent on Acyl-CoA synthetase long-chain family member 4 (ACSL4) through shaping the cellular lipid composition to a great extent. In the mechanism, ACSL4 contributes to the formation of cell membranes rich in long polyunsaturated ω6 fatty acids that are easily attacked in ferroptosis process [41, 42].

Studies have revealed that ferroptosis is also an autophagy-dependent cell death pathway [43]. Hou et al. have found that autophagy can deplete ferritin in tumor cells and fibroblasts to promote the induction of ferroptosis [44]. Besides, due to the ability of lipid droplet to prevent PUFA from peroxidation, its degradation mediated by lipophagy can promote lipid peroxidation in ferroptosis [43]. On the other hand, the detoxication of lipid peroxidation against ferroptosis depends on the activity of glutathione peroxidases (GPX) [45, 46]. GPX4 is a type of GPX enzyme that can reduce peroxidized phospholipids inserted into membranes, it will be inactivated when intracellular glutathione (GSH) level decreases. Similarly, the synthesis of GSH could be hindered when the xc cystine/glutamate antiporter system is under blockade [46–48]. Therefore, a decreased GPX4 expression is relevant to an increased ferroptosis sensitivity. The abnormal function of a cystine-glutamate antiporter (system Xc-) is also important for ferroptosis execution [49].

Ferroptotic tumor cells can induce immune response by releasing HMGB1 [50, 51]. HMGB1 is an essential DAMP and exerts a significant effect in the immunogenicity of dying tumor cells. ATP and CRT have also proven to be the crucial DAMPs in the process of ferroptosis [51, 52]. Moreover, decorin has been identified as an important DAMP on account that it can act on the advanced glycosylation end-product-specific receptors on macrophages, triggering the generation of pro-inflammatory factors and eliciting inflammatory and immune response though ferroptosis [53]. Krysko’s research team first demonstrated the immunogenicity of ferroptosis [54]. They found that the ferroptosis of murine fibrosarcoma MCA205 cells could increase the number of BMDCs with mature phenotype and exert a vaccine-like action in vivo, which suggested that ferroptosis could trigger an innate and adaptive immune response.

Interestingly, other evidences for immunogenicity of ferroptosis have been found in a cardiac injury model, the researchers found that ferroptotic cells can attract neutrophils by activating Toll-like receptor 4 (TLR4)-TIR domain containing adaptor inducing interferon beta (TRIF) [55]. In addition, Immune cells activated by ferroptosis promote the induction of tumor cells ferroptosis in turn by secreting cytokines [56]. For example, IFNγ produced by cytotoxic T lymphocytes (CTLs) can activate the Janus tyrosine kinase (JAK) signal and signal transducer and activator of transcription 1 (STAT1) pathway. It can also downregulate the level of endogenous SLC7A11 and SLC3A2, which are two subunits of cysteine/glutamate anti-transporter system Xc − , suppressing the Xc-system function, increasing intracellular stored iron content, hindering the endogenous production of GSH and triggering lipid peroxide of the tumor cells thereby inducing ferroptosis [56]. Likewise, transforming growth factor-β (TGF-β1) secreted by macrophages could promote ferroptosis by suppressing the Xc-system function via SMAD signaling [57].

All of these studies powerfully confirm the immunogenic potency of ferroptosis and indicate that it could be applied in cancer therapy. However, while there is evidence suggesting that ferroptosis may have a synergistic effect on antitumor immunity, there are theoretical inconsistencies that require further research. One of the theoretical concerns is that cancer cells undergoing ferroptosis may potentially serve as donors of arachidonic acid (AA) for the transcellular biosynthesis of eicosanoids. This process could lead to the production of biologically active immunomodulatory AA metabolites that could impact tumor immunotherapy [36]. Additionally, accumulating evidence indicates that increased intratumor generation of prostaglandin E2 (PGE2) can facilitate the evasion of immune surveillance by tumors [58]. The induction of ferroptosis in tumor cells has been associated with the release of PGE2 [46]. Therefore, the production of PGE2 may present an inherent obstacle to eliciting a robust immune response by ferroptotic cells. In essence, the relationship between ferroptosis and antitumor immunity, there are complexities and theoretical challenges that warrant further investigation.

As a key component of ferroptosis, Reactive oxygen species could induce lipid peroxidation that can have significant implications for the modulation of immunity in human malignancies, in addition to its role in oxidative stress [59]. Elevated ROS levels can lead to T cell exhaustion and inhibit the activation and proliferation of T cells, contributing to tumor immune evasion. ROS can also inhibit the formation of T-cell receptor and MHC antigen complexes in T cells, thereby suppressing antitumor immunity. Accordingly, scavengers of ROS can enhance the activation of cytotoxic T lymphocytes by activating superoxide dismutase 2 (SOD2) [60, 61]. The ability of CAR-T cells to eliminate tumor cells has been associated with lower levels of intracellular oxidative stress. Oxidative stress or ROS can also have an impact on regulatory T cells, promoting their immunosuppressive function [62]. Myeloid-derived suppressor cells (MDSCs), which are induced by tumors, can also inhibit T cell proliferation and promote tumor growth by producing ROS. However, the negative effects can be counteracted by catalase, an enzyme that reduces ROS levels. This, in turn, restores the action of T cells [63]. Overall, the interplay between ROS and immune cells within the TME is a complex and critical aspect of cancer immunology. Managing oxidative stress and its impact on immune cells may be helpful for improving cancer immunotherapy strategies. Hence, there are still urgent needs to exploit new therapeutic approaches based on ferroptosis.

### Pyroptosis in antitumor immunity

Pyroptosis, a caspase-dependent programed cell death, is involved in inflammation and mediated by a family protein called gasdermins (GSDMs) [64]. Pyroptosis features DNA fragmentation and chromatin condensation accompanied by cell swelling, capture, and the discharge of many pro-inflammatory cytokines, such as IL-1β, IL-18, HMGB1 and ATP, caused by the formation of transmembrane pores due to GSDM cleavage, leading to increased intracellular osmotic pressure and eventually cell membrane rupture [65, 66].

Activation of pyroptosis in dying cells relies on the following two major pathways: (I) GSDMD-dependent pyroptosis modulated by caspase-1/4/5/11; (II) GSDME-dependent pyroptosis modulated by caspase-3 [67–71]. Caspases-1 and-11 as well as the apoptosis effector caspase-3 could induce different but interwoven pyroptotic cell death based on the activation of GSDMs, which is important during the pyroptosis process due to its potential membrane punching activity [68, 72–74]. Activated caspases-1 can cleave GSDMD and produced gasdermin-C and gasdermin-N domains. Activated caspase-3 cleave GSDME. They both can release the fragment with lethal activity and lead to pyroptosis [75, 76]. To be specific, the gasdermin-N domain contributes to the generation of transmembrane pores that connect cytosol to extracellular matrix. Consequently, the membrane potential and cellular homeostasis change due to potassium efflux and water influx, causing cell swelling. Further cell lysis and capture causes the release of numerous inflammatory cytokines and cellular contents, thus activating an intensive local inflammatory response [65, 66].

Pyroptosis is thought to be highly immunogenic because of its ability to promote the release of pro-inflammatory cytokines and DAMPs, which could boost the stimulation of both intrinsic and adaptive immunity response [64, 77, 78]. For instance, activation of GSDME in pyroptosis process could restrain tumor growth through promoting the recruitment of CD4^+^ and CD8^+^ T lymphocytes and NK cells with antitumor properties [78]. Moreover, a recently published study conducted by Zhang et al. indicated that a positive feedback loop could been established in the immune microenvironment activated by pyroptotic cell death, for which CD8^+^ T cells and NK cells could in turn trigger cancer cells pyroptosis by granzyme B that is able to cleave GSDME. They also reported that 20 of the 22 tested cancer-associated GSDME mutations reduced the function of GSDME, a strategy that tumor cells escape the attack of immune system through inactivating GSDME [20].

Up to date, it has been found that proapoptotic caspase-3 can also cleave GSDMs in cells abundant with GSDMs [64, 77, 78]. On account of the link between GSDMs and apoptotsis, the increasement of these intracellular proteins can promote the transition of immunosilent apoptosis to immunostimulatory pyroptosis. Therefore, it could be considered as a new and remarkable strategy that may eliminate the tolerance of most tumor cells to apoptosis by means of converting apoptosis to pyroptosis, eventually improving cure rate, reducing metastasis and relapse thanks to the activation of the adaptive immune system [79].

### Autophagy in antitumor immunity

Autophagy, a cellular regulatory mechanism that eliminates redundant or malfunctioning cellular components and recycles metabolic materials, plays a pivotal role in the tumor microenvironment. Stress signals within this environment lead to alterations in autophagy pathways in both tumor and immune cells, resulting in diverse effects on tumor progression, immunity, and treatment. Autophagy influences the survival and apoptosis of immune cell subpopulations, their differentiation, activation, effector function, and their migration to the tumor site. Simultaneously, tumor-autonomous autophagy can modify tumor growth by impacting the immune response. Consequently, autophagy represents a complex yet promising target in cancer therapy.

In response to various stress states, autophagy occurs in the cell. Specifically, as a metabolic process, autophagic membrane structures are formed inside cells where they can recognize damaged organelles, unfolded proteins and pathogens through selective autophagic receptors (SARs), and these intracellular structures are subsequently phagocytosed and degraded [80, 81].

Eukaryotic cells maintain homeostasis and manage lipid metabolism by autophagy which is vital for cell survival. The autophagy initiation depends on the active state of the unc-51-like kinase (ULK) complex [82], the activation of which occurs when stress signals stimulate 5′-AMP-activated protein kinase (AMPK), or mTOR complex 1 (mTORC1) is suppressed, vacuolar protein sorting (VPS34) is subsequently activated to generate phosphatidylinositol 3-phosphate (PI3P) and recruit PI3P-binding molecules [83, 84], resulting in the generation of a separated pre-autophagosomal structure called phagosome [85, 86]. The receptor on phagosome combines with specific cargoes via ubiquitin labeling, which is a critical process in autophagy for the selective recruitment of loads [87–90]. Then the phagosome continuously expands and finally closes, converting to autophagosome. Transited to the perinuclear region, autophagosomes integrate into proximal lysosomes and shift to autolysosomes. Finally, cargoes will be decomposed, and nutrients will be recycled by lysosomal hydrolases [91–93].

Studies have shown potential relationships between autophagy and tumor immunity responses, including inherent immunity, antigen presentation, and inhibition of immune evasion, suggesting its fundamental role in multiple immune responses [94]. For instance, autophagy could boost the secretion of adenosine triphosphate (ATP) by promoting the move of lysosomes loaded with ATP towards the plasma membrane [95]. As a “find-me” signal, ATP is crucial for stimulating the tumor infiltration of cytotoxic T lymphocytes. What’s more, autophagy could also contribute to antigen process and presentation. Tumor cytoplasmic constituents are engulfed by autophagosomes for the processing of endogenous antigens, which are presented on the surface of APCs, stimulating CD4^+^ T cells [96]. Studies have showed that when ATG5 is defective, the formation of autophagosomes will be delayed, consequently affecting antigen delivery by dendritic cells (DCs) via the MHCII [97, 98]. Autophagy can facilitate the presentation of extracellular antigens to major histocompatibility complex II (MHCII) through an atypical autophagy pathway known as ATG8/LC3-related phagocytosis (LAP). LAP plays a role in the process of macrophages engulfing and breaking down dying cells, leading to the presentation of antigens to immune effector cells [99]. Besides, an increased number of evidences indicate that autophagy may degrade immune checkpoint protein to suppress tumor cells’ immune escape. As we all know, PD-1/PD-L1 ICBs have been broadly applied in cancer immunotherapy by hindering the link between PD-L1 on cancer cells and PD-1 on T cells [100, 101]. A recent study has concluded that Huntingtin-interacting protein 1-related (HIP1R), an autophagy receptor, can trigger PD-L1 degradation in lysosomes by binding with PD-L1, activating T cells and suppressing the tumor growth [102].

However, autophagy is a double-edged sword which may not only enhance but also suppress the development, maturation and normal physiological function of immune cells [103–105]. Treg cells can inhibit antitumor immune responses by inducing autophagy. For example, Treg cells in human melanoma inhibit the activation of arginine-mediated mTOR by expressing larger numbers of arginase 2 (ARG2), which can degrade endogenous arginine, and then induce autophagy [106]. In addition, cancer cells can achieve immune evasion through the degradation of MHC I complexes by selective autophagy [107, 108]. A case in point, in pancreatic cancer cells, the MHCI complexes could be delivered to the endo-lysosome and degraded via ubiquitin-binding receptor NBR1, leading to the failure of recognition by T cells and resistance to ICBs [109]. In contrast, suppressing autophagy helps to restore the levels of MHCI complexes and enhances antigen presentation. Thus, autophagy plays a significant role in helping tumors evade immune surveillance by CTLs, leading to the development of immune tolerance. Research has shown that the autophagy induced by the 5-hydroxytryptamine/5-hydroxytryptamine 1a receptor (5HT/5-HT1aR) signaling pathway contributes to the creation of an immunosuppressive environment in non-small cell lung cancer (NSCLC). This autophagy leads to tumor cell resistance to CTL-mediated lysis through the phosphorylation of STAT3 [107]. Further research shows that autophagy-deficient host mice tumor models tend to have increased infiltration of immune cells compared to those with intact autophagy, which can be attributed to the activation of the STING pathway [110]. Furthermore, autophagy activation in the liver's TME can also create an immunosuppressive setting that hinders the innate immune response and subsequently limits the antitumor activity of T cells. It is essential of specific deletion of autophagy in liver hepatocytes for inducing tumor rejection [110]. Collectively, these findings suggest that autophagy process, mainly through the degradation of MHCI/II complexes, in both tumor cells and immune cells, can facilitate tumor immune escape and contribute to immune tolerance.

The interplay between oncogenes and autophagy genes in tumorigenesis has been further studied. There are over 40 genes encoding autophagy-related proteins (ATGs) involved in the autophagy pathway [111]. Mutations in ATG-encoding genes may contribute to tumor initiation and impact immune system recognition [112]. Research has shown that mosaic deletion of Atg5 or Atg7 in the mouse liver leads to benign hepatomas, indicating that complete and specific autophagy deficiency can promote the initiation of liver tumors but limits their progression to malignancy [113]. A similar phenomenon has been observed in KrasG12D-driven pancreatic cancer models [114, 115]. In the context of BrafV600E-driven melanoma, Atg7-deleted tumors exhibit enhanced oxidative stress and senescence, halting further tumor progression. This shows that autophagy, by reducing oxidative stress and overcoming senescence, promotes BrafV600E-driven melanoma [116]. Additionally, autophagy defects may indirectly promote tumorigenesis through inflammation [117]. Mice with ATG16L1 deficiency are prone to acute colitis induced by dextran sulfate sodium [118, 119]. Interestingly, Atg16l1T300A knock-in mice display similar impaired antibacterial host defense, leading to chronic inflammation, tissues damage, and an increased cancer risk [120]. In summary, a mild or intermediate deficiency and activation in tumor cells autophagy can facilitate tumorigenesis and promote cancer progression, while complete loss or stimulation of autophagy can trigger tumor cell senescence, impeding cancer progression.

Based on the above discussion, autophagy exerts important function in tumor antigen processing and presentation, as well as immune cells recruitment, but autophagy activation can also facilitate tumor cells escape from immune surveillance, causing innate resistance to cancer immunotherapy. Furthermore, while there is substantial evidence supporting the significance of autophagy in various conditions such as cancer, neurodegenerative diseases, and infectious diseases, it's important to note that, as of now, no specific autophagy inhibitor or inducer has gained regulatory approval for use in cancer or any other disease [121]. This underscores the necessity of developing improved pharmaceuticals for targeting autophagy.

## The application of nanomaterials-induced ICD in antitumor therapy

### Nanomaterials-based photothermal therapy

As a form of phototherapy, photothermal therapy (PTT) usually employs optical absorbing agents to selectively kill cancer cells. In the PTT process, the tumor sites enriched with photothermal conversion reagents (PTAs) are irradiated with laser to convert light energy into thermal energy [122, 123]. Especially in the near infrared (NIR) biological window (700 ~ 1400 nm), PTT can achieve deep tissue penetration, reduce the thermal effect of tissues and thus reduce the light damage to adjacent healthy organs and tissues [124–127]. When the tumor is heat treated, some heat- sensitive cell proteins will denature and then form polymers with other proteins in the cancer cells [128, 129]. Protein denaturation and aggregation caused by overheating will seriously damage some physiological activities in the body, such as protein (enzyme) inactivation, chromatin change, inhibition of DNA synthesis and repair, and ultimately lead to cancer cell death [130, 131].

However, the application of PTAs in clinic is greatly limited by their disadvantages, including photobleaching, nonspecific intracorporeal distribution, and short circulation time. When drug transport systems based on nano-technologies are combined with PTT, the range, time and efficiency of therapy could be accurately modulated by altering the location, duration and power of irradiation [132, 133]. Therefore, it is of great significance for antitumor treatment to develop efficient and safe nano-PTAs. At present, nano-PTAs, such as noble metal materials, metal sulfur compounds, two-dimensional materials, organic small molecules and semiconductor polymers have been applied in the field of antitumor PTT [134–137]. For example, several metallic nanocarriers represented by gold nanoparticles have been reported for their photothermal properties, of which could be modulated by the alteration in morphology and size [138, 139]. In addition, NIR-responsive nanocarriers have also been prepared through the combination with small molecules such as indocyanine green (ICG) and Infrared dye (IR780) [140, 141].

More importantly, photoactivated ICD is a feasible treatment approach for tumor cell ablation under the irradiation of the NIR. NIR-responsive nanoparticles may stimulate the discharge of DAMPs and some other costimulatory molecules to improve cellular immunity. In addition to the secretion of DAMPs, the enhancement of the lymphatic circulation during photothermal therapy can stimulate the migration of T cells and APCs due to temperature rise [142, 143]. Zhang et al. [144] have developed a magnetic transportation system for efficient tumor therapy via PTT. In their study, R837, an immune adjuvant as Toll-like-receptor-7, was co-loaded with ICG in this delivery system. When the nanoparticles accumulated at tumor sites, tumor-associated antigens (TAAs) were released upon NIR light irradiation and immune responses were further boosted by R837. In general, compared with traditional treatment methods, PTT based on nanomaterials mediated by external laser stimulation has greater advantages [145–148].

### Nanomaterials-based photodynamic therapy (PDT)

Photodynamic therapy is also a new cancer therapeutics, which can be applied to the clinical treatment of various surface tumors [149–151]. PDT usually uses photosensitizers to kill tumor cells by shifting the surrounding oxygen molecules to ROS under the appropriate light irradiation, including singlet oxygen (^1^O_2_), superoxide anion (·O_2_^−^), hydroxyl radical (·OH) and hydrogen peroxide (H_2_O_2_) [152, 153]. The inherent tumor selectivity of photosensitizers and the combined localization effect of light make photodynamic therapy more accurate and less traumatic [154]. At the same time, photodynamic therapy mechanism based on production of reactive oxygen can reduce systemic toxicity and avoid causing resistance to tumor drugs [154].

However, traditional photosensitizers have poor water solubility, short excitation wave and can only treat superficial cancer. The quantity and performance of photosensitizers are still difficult to meet the needs of clinical applications [155]. Therefore, the development of photosensitizers based on nanomaterials has attracted much attention. Compared with traditional photosensitizers, nano photosensitizers have more promising photophysical properties, easier functionalization and better tumor treatment effect. What’s more, they can easily penetrate and remain in tumor tissues, achieving passive tumor targeting [156].

For example, Chlorine e6 (Ce6), a natural chlorophyll derivative, has been broadly applied for PDT, but its application is restricted because of the absence in tumor sites. Chen et al. [157] exploited a hybrid protein oxygen nanocarrier based on protein hybridization, which was loaded with chloramphenicol e6 to mediate oxygen-enhanced immunogenic PDT. This nanoparticle can co-transport Ce6 and oxygen into cancer cells, greatly relieving the hypoxia. The effect of PDT was consequently augmented and then ICD was induced in tumor cells. There are also some investigations about the combined treatment of PTT and PDT. Li et al. [158] presented a dual ER-targeting approach to achieve PDT, PTT and immunotherapy. This thought is based on the theory that ROS produced via ER stress can induce ICD. They constructed hollow gold nanospheres (HAuNS) to trigger severe ER stress, which were conjugated with ICG and modified by ER-targeting pardaxin (FAL)-peptide, together with an oxygen-delivering hemoglobin liposome (FAL-Hb lipo). The approach successfully improved the CRT exposure and stimulated systematic immune responses, enhancing the efficacy of immunotherapy in cancer treatment.

Above all, Nanoparticle-based physical therapies can not only effectively kill cancer cells but also promote ICD induction to activate the antitumor immune response, suggesting their tremendous potentials in the field of cancer treatment.

### Delivery of immunogenic cell death inducers by nanomaterials

Drug delivery systems based on nanoparticle can improve significantly the tumor targeting ability of physical cues and promote the antitumor efficacy, this method is applicable equally to the transportation of many chemotherapeutic agents or ICD inducers. According to their ability of either activating the cell death phenomenon or the discharge of DAMPs, ICD inducers fall under two categories, including type I (ROS inducers) and type II (ER inducers) [159, 160]. The most of chemotherapeutic agents belong to type I with the ability of inducing apoptosis, containing natural compounds represented by doxorubicin (DOX) or mitoxanthrone and synthetic drugs represented by oxaliplatin (OXA) or cyclophosphamide [161–164]. Chemotherapeutic agents can activate ICD in different ways, including promoting the production and release of DAMPs to enhance tumor cell immunogenicity, or realizing the increasement of TAAs expression and enhancement of antigen presentation, and recruiting immune cells towards tumor sites [165, 166]. Type II inducers, including coxsackievirus B3 and some PDT strategies, could lead to disturbances in endoplasmic reticulum (ER) homeostasis and induce ICD [79, 167, 168].

**Fig. 2:**
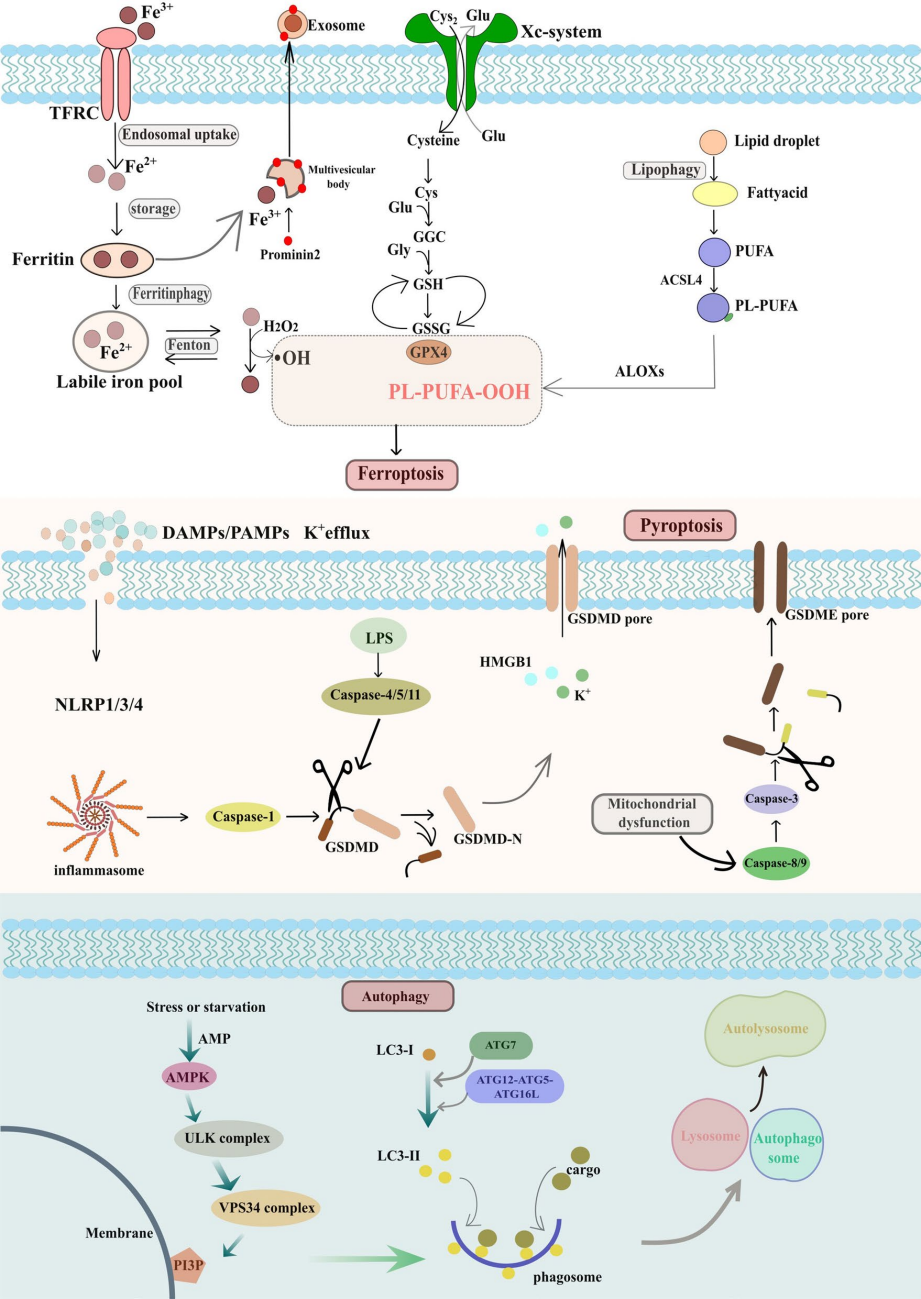
The simplified mechanism of ferroptosis, pyroptosis and autophagy

However, the application of ICD mediated by chemotherapeutic agents is limited significantly due to their short half-lives in circulation, degradation by enzymes, and off target effects [169, 170]. To address this problem for better cellular immunity, nanoparticles have been used as a promising delivery carrier of various chemotherapeutic drugs needed to be transported precisely toward the tumor tissue [139, 171, 172], for which nanoparticles can co-deliver the cytotoxic drugs, photosensitizers and immunoadjuvants to the cancer sites and enhance their permeation and retention effects [173]. For example, Qi et al. [174] constructed an innovative chemo-immunostrategy, they developed AEAA-polymer-disulfide-bond nanoparticles to deliver two medicines, mitoxantrone (MIT) and celastrol (CEL), thanks to their the excellent antitumor and antifibrosis potentials. As a tumor-targeting molecule, AEAA worked synergistically with MIT and CEL to trigger ICD and promote TAA recognition by immune cells for the initiation of systemic immune responses. The study demonstrated that the nanoparticles successfully remodeled immune inhibition microenvironment in tumor site, effectively inhibiting tumor growth and metastasis (Fig. 2).

Collectively, Nanoparticle-mediated chemotherapy improves the tumor targeting and biocompatibility of chemotherapeutic agents, while being able to effectively induce ICD to activate antitumor immune responses, achieving brilliant therapeutic benefits. Thus, nanodrug delivery systems could have a significant effect in tumor chemotherapy-immunotherapy.

## The application of nanoparticles-based ferroptosis, pyroptosis and autophagy in cancer therapy

### The application of nanoparticles-based ferroptosis in cancer therapy

#### Iron-based nanoparticles

Iron-based nanomaterials represented by iron oxide nanoparticles (IONPs) and iron-organic frameworks have achieved broad application in tumor therapy thanks to their ability of inducing ferroptosis [175, 176]. In addition to being used for magnetic resonance imaging (MRI) as contrast agents, they can catalyze Fenton reaction to accelerate reactive oxygen species (ROS) production that is crucial to ferroptotic cell death [177–179]. The majority of iron-based nanoparticles depend on the discharge of Fe^2+^ to activate the Fenton reaction [180, 181]. However, due to the poor ROS conversion efficiency of Fe^2+^ in TME (pH 5.5 − 6.5), iron-based nanoparticles are often combined with other treatment strategies for a better synergistical effect [182–186].

**Fig. 3:**
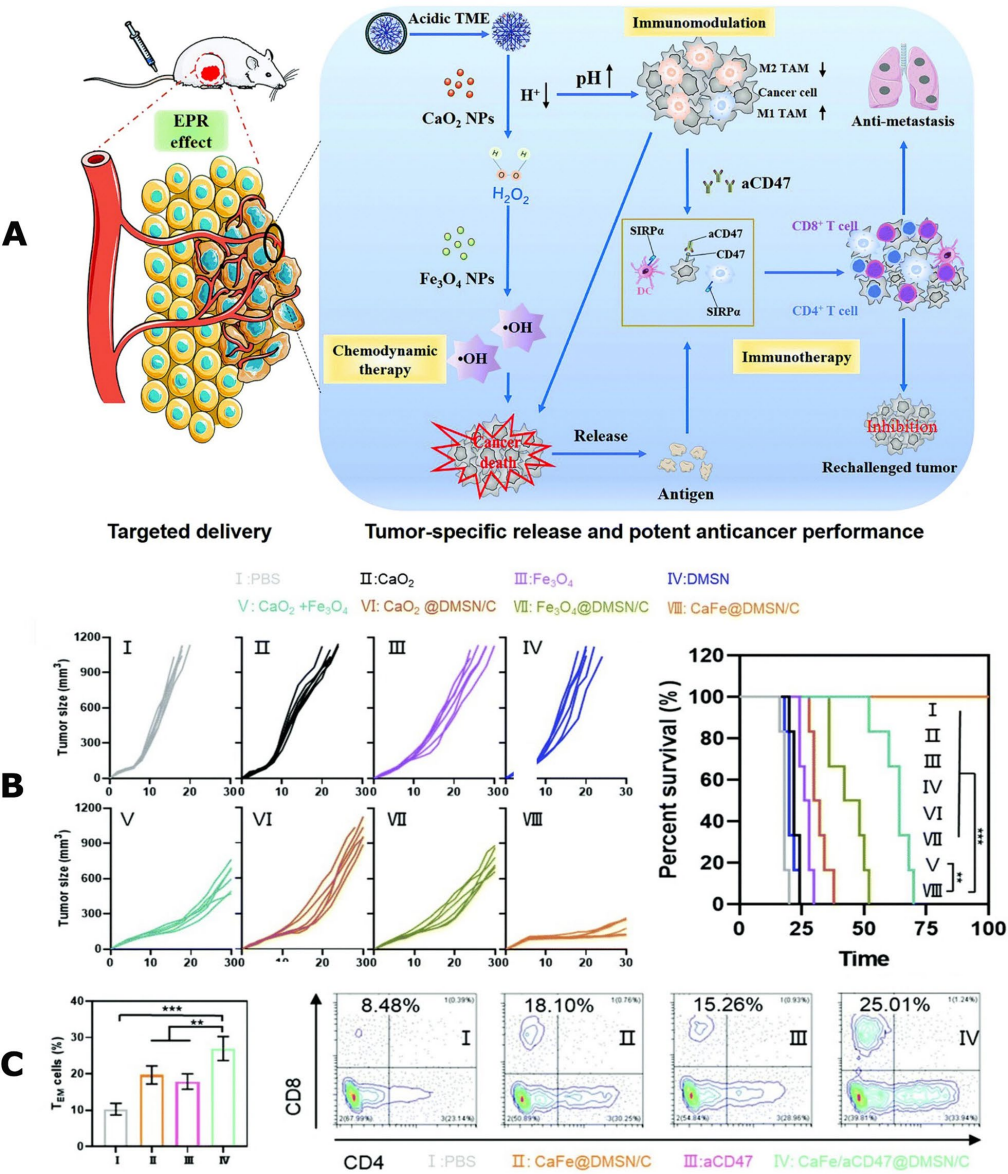
**A** Schematic illustration of the cascade reaction-mediated efficient ferroptosis synergized with immunomodulation/immunotherapy for high-performance tumor ablation. **B** Individual tumor growth kinetics and survival of mice of the primary tumors receiving different treatments. Data are represented as mean ± SD (n = 6) **C** Percentage of TEM cells in the spleens of the mice with different treatments on the same day of rechallenging and Representative flow cytometric analysis of CD8 + T cells gating on CD3 + cells in the distant tumors on day 30. Data are represented as mean ± SD (n = 6). Copyright 2020, ROYAL SOC CHEMISTRY publishing group

The Fenton reaction mentioned above is an interaction between hydrogen peroxide and ferric ions, leading to generation of hydroxyl radicals that could cause lipid peroxidation. Accordingly, it is a desirable approach to catalyze the Fenton reaction in tumor cells via intratumor drug delivery [187, 188]. For example, Li and Rong et al. [189] developed an efficient nano-catalytic formulation coated with a pH-responsive membrane (DMSN NPs) (Fig. 3). Based on a cascaded reaction, ultrasmall CaO_2_ and Fe_3_O_4_ NPs were encapsulated into DMSN NPs, this strategy not only avoided the leakage of drugs, but also achieved tumor-targeting accumulation. In their study, the DMSN NPs were intravenously injected in 4T1tumor-bearing mice. The cascade reaction was triggered by the H^+^ ions reacting with CaO_2_ in a weak acidic microenvironment and producing a great deal of H_2_O_2_ in the tumor site. Subsequently, mediated by Fe_3_O_4_ NPs, the generated H_2_O_2_ was catalyzed into cytotoxic hydroxyl radicals (·OH) via a Fenton-like reaction and triggered ferroptosis process, promoting TAAs release and establishing an immunogenic TME. Similarly, Zhang et al. [190] designed a dual-homeostasis nanoparticle (MOF@GOx@MnO_2_@PEG: MGMP) modified by polyethylene glycol (PEG) on iron-based metal–organic framework (Fe-MOF), as well as loading the MnO_2_ and glucose oxidase (GOx). Their results demonstrated that the disruptor achieved intensive ferroptosis induction and immunotherapy via the continuous accumulation of iron ions and H_2_O_2_ in cancer cells by means of inhibiting H_2_O_2_ clearance, promoting H_2_O_2_ production, and restraining iron ion efflux. In addition, they combined the disruptor with Apt-PD-L1 checkpoint blockade able to prevent the immune escape, greatly promoting the ferroptosis-based antitumor therapy efficiency. Previous researches have explored methods involving the delivery of excess Fe^2+^/Fe^3+^ ions to elevate intracellular iron concentrations as part of ferroptosis-based therapy [191–193]. However, the robust homeostasis of intracellular iron metabolism continues to present challenges for the success of these therapeutic strategies. For example, Ferroportin 1 (FPN1) plays a crucial role in regulating iron homeostasis by transporting excess iron out of cells, preventing the accumulation of large quantities of iron [194]. Different strategies have been devised to modulate iron metabolism [195]. Nevertheless, depleting GSH or increasing H_2_O_2_ levels alone may not effectively disrupt the redox homeostasis. Even when an ample supply of H_2_O_2_ is generated, the labile iron pool within cancer cells can only provide a restricted quantity of iron ions, presenting a significant obstacle for ferroptosis-based cancer therapy. Therefore, it is prudent to design a potent dual homeostasis disruptor that can effectively disrupt both intracellular redox balance and iron metabolism homeostasis. This innovative approach presents a promising method for ferroptosis-based immunotherapy of cancer.

Iron-based nanoparticles could also work synergistically with PDT by means of the ROS photoinduction. Chin et al. [196] developed cluster-structured nanoparticles (CNPs) that consisted of Fe_3_O_4_ and iron chlorophyll (Chl/Fe) photosensitizers. Their results demonstrated that Fe-based nano-photosensitizers could activate the Fenton reaction based on chemodynamic therapy (CDT), result in depletion of GSH and GPX4. In addition, CNPs promoted the induction of ferroptosis combined with PDT. This CDT–PDT therapy strategy obviously suppressed cancer progression and remodeled the tumor immune microenvironment (TIME) into an immunostimulatory microenvironment. This study introduced a novel multifunctional nanoparticle agent that served multiple purposes: 1. Enhanced Delivery Efficacy: It improved the delivery efficiency, allowing for a broader distribution through minimally invasive therapeutic techniques. 2. Redox Balance Disruption: It incorporated lipid peroxidation activator adjuvants to disrupt redox balance through the induction of ROS. 3. Combined Photodynamic and Chemodynamic Therapy: It loaded nano-photosensitizers for a combination of PDT and CDT, which collectively aimed to reprogram the TME and mitigate the immune escape effect. This study suggested that the integration of PDT with other endogenous chemical therapeutic strategies is intended to enhance the effectiveness of treatment for malignant cancer. While these designed nanoagents represent promising advances, they are complex and leave room for further refinement and improvement.

In general, these studies proved that Iron-based nanoparticles could break the dual homeostasis of redox and iron metabolism by supplying H_2_O_2_ and downregulating GSH or GPX4 to increase the level of intracellular lipid peroxidation, contributing to ferroptosis of cancer cells as well as the tumor infiltration of immune cells. Ferroptosis-mediated cancer treatment is probably a promising method.

**Fig. 4:**
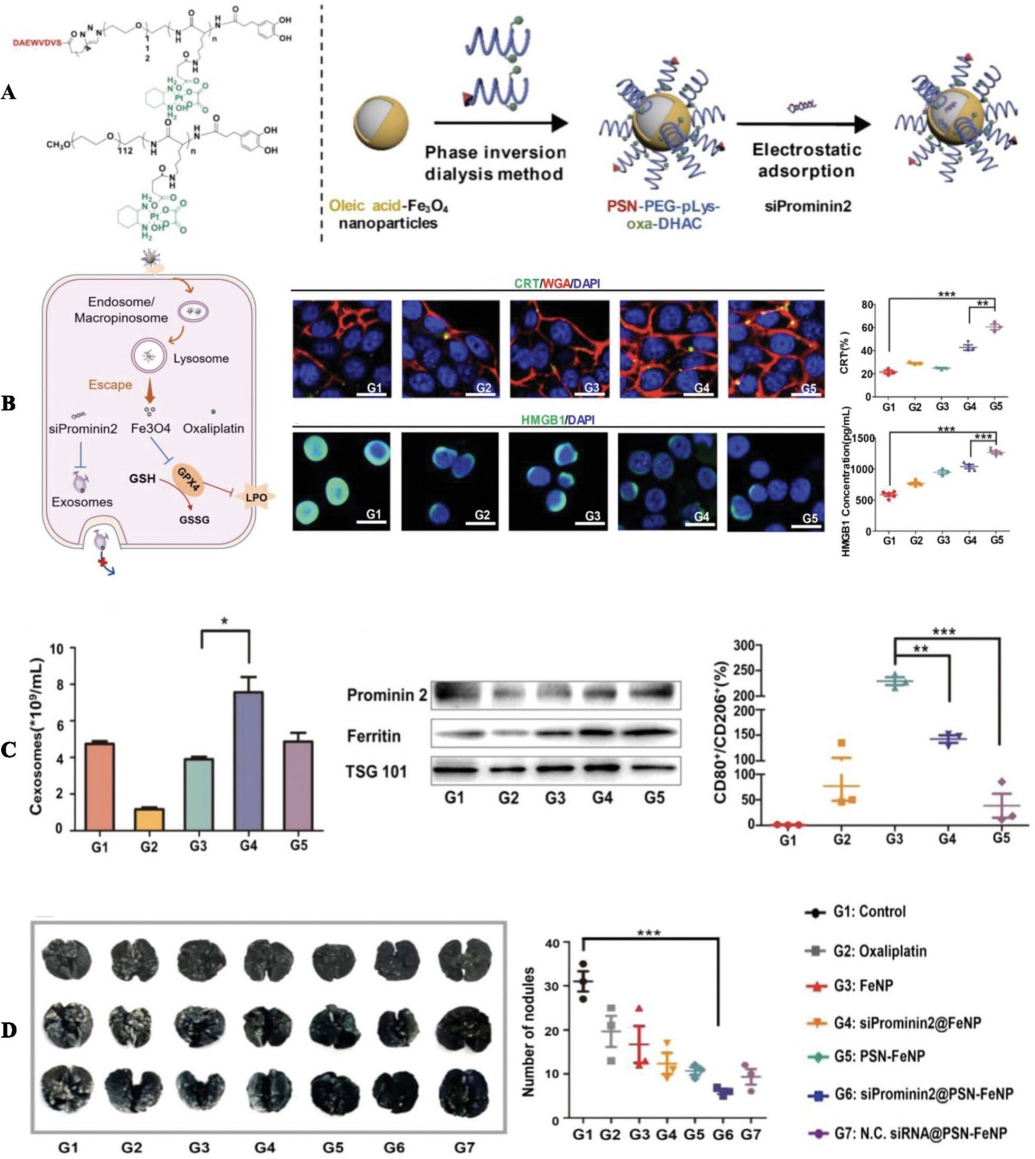
**A** Fabrication of the siProminin2@PSN-FeNP **B** Illustration of ferroptosis induced by siProminin2@PSN-FeNP and CRT exposure and HMGB1 secretion in 4T1 tumor cells, following by CLSM (scale bars = 20 µm) G1: Control; G2: Oxaliplatin; G3: FeNP; G4: PSN-FeNP; G5: siProminini2@PSN-FeNP. **C** Number of 4T1 tumor cells-derived exosomes quantified with NTA, respectively (n = 3) and western blot assay of Prominin2, Ferritin expression in 4T1 tumor cells-derived exosomes (left); Flow cytometry analysis results of CD206 and CD80 expression in RAW 264.7 (pretreated with IL-4), respectively (n = 3) (right) G1: Control; G2: siProminin2; G3: siProminini2@PSN-FeNP; G4: PSN -FeNP; G5: iron ions. **D** Number of the lung metastatic foci and its quantification, respectively (n = 3). Data were presented as the mean ± SD (one-way ANOVA comparisons tests, ∏P < 0.05, ∏∏P < 0.01, ∏∏∏P < 0.001). Copyright 2022, WILEY publishing group

However, not all Iron-based nanoparticles rely on the Fenton reaction to trigger ferroptosis. Some studies attempted to induce ferroptosis by interfering biological process of tumor cells. For example, exosomes have been shown to inhibit ferroptosis process in tumor cells because they can deliver iron extracellularly when the intracellular iron concentration is excessively high [197–199]. Wang et al. [200] proposed a triple therapy by developing a hybrid nanoparticle(siProminin2@PSN-FeNP), which was composed of a biocompatible oleic acid-Fe_3_O_4_ core, oxaliplatin and Prominin2 siRNA (Fig. 4). According to their investigation, the siProminin2 can mediate exosomal inhibition. PSN peptide was utilized to modify nanoparticles, which allowed them to target cancer cells combined with oxaliplatin-mediated ICD. Their results demonstrated the combination of oxaliplatin, ferroptosis induction and exosomes inhibition can synergistically augment the antitumor immune responses on Orthotopic Breast Cancer Model. They also showed the tremendous potential of the combined application of ferroptosis-mediated cancer treatment and immunotherapy, and indicated that inhibiting the release of exosomes could play a positive role in the antitumor therapy. An effective strategy for inducing ferroptosis, as previously mentioned, involves increasing intracellular iron content. However, for valid ferroptosis-based tumor treatments, a relatively high iron content is often required, sometimes as much as 75 mg/kg [201]. Nevertheless, excessive iron can lead to serious systemic toxicity, even exacerbate cancer malignancy, and promote metastasis [201]. Thus, the challenges of ferroptosis-based cancer immunotherapy lie in using iron as a ferroptosis inducer without triggering unforeseen adverse effects. Study has revealed a mechanism for exporting iron to maintain iron homeostasis in tumor cells [197]. In situations where cells are susceptible to ferroptosis, a pentaspan membrane glycoprotein known as Prominin2 can mediate intracellular iron efflux through tumor cell-derived exosomes, thereby resisting ferroptosis. As this article showed, it would be beneficial to design nanoplatforms with anti-exosomal iron efflux properties that can simultaneously improve the antitumor immunity.

Apart from the above approach, nanoparticles can trigger ferroptosis process by inhibiting intracellular signaling pathway. Hsieh et al. [202] have reported a zero-valent-iron nanoparticle (ZVI-NP), which could inactivate NRF2-mediated cytoprotective program. The mechanism is involved in the GSK3β/β-TrCP-dependent depletion of NRF2 by activating AMPK/mTOR pathway. Their study proved that ZVI-NP successfully caused ferroptosis of lung cancer cells. Moreover, ZVI-NP enhanced antitumor immunity by reducing the number of Tregs, converting M2 macrophages to M1 macrophages, decreasing the level of PD-1 and CTLA4 in CD8^+^ T cells while attenuating PD-L1 expression in tumor cells. They have discovered a new mechanism wherein ZVI-NP promotes the phosphorylation-dependent ubiquitination and degradation of nuclear factor-E2-related factor 2 (NRF2), which is a critical transcription regulator responsible for maintaining cellular redox homeostasis. By inducing excessive oxidative stress and lipid peroxidation, this mechanism triggers ferroptotic cell death. This research holds significant importance in the development of anti-cancer strategies that aim to induce ferroptosis with greater efficacy while ensuring improved safety. Similarly, it is well known that the generation of GSH depends on xc cystine/glutamate antiporter system. Thus, Jiang et al. [203] constructed a sulfasalazine (SAS)-loaded magnetic nanoplatform (Fe_3_O_4_-SAS@PLT) modified with platelet (PLT) membrane. This nanoplatform can induce ferroptosis in tumor sites by inhibiting the glutamate-cystine antiporter system X_c_^−^ pathway. It also triggered an intensive immunological response and promoted the therapeutic effect of PD-1 blockade in vivo. In addition, their study also indicated that Fe_3_O_4_-SAS@PLT-induced ferroptosis can repolarize tumor-associated macrophages from M2 phenotype to M1 phenotype. The design incorporated Fe_3_O_4_ nanoparticles as ferroptosis inducers, which can synergistically work in conjunction with SAS and consequently reduced dosage of SAS. Furthermore, the platelet membrane coating confers immune evasion and tumor targeting capabilities to Fe_3_O_4_-SAS@PLT. Given the crucial role of iron in ferroptosis, many studies have concentrated on iron-based nanomaterials for cancer treatment, including ferumoxytol [204], inorganic iron nanoparticles [205], and iron-organic frameworks [184]. These nanomaterials have shown potential in enhancing the effectiveness of ferroptosis in cancer treatment. However, clinical applications have been limited due to the lack of immune evasion and poor tumor targeting. To address this issue, cell membranes derived from entities like red blood cells, platelets, and macrophages have been employed to camouflage nanomaterials, helping them evade immune clearance [206, 207]. Consequently, cell membrane-coated nanoparticles with immune evasion and tumor-targeting capabilities have the potential to maximize the delivery of ferroptosis-inducing nanoparticles to tumors. This approach holds promise in the context of ferroptosis therapy.

#### Iron-free nanoparticles

**Fig. 5:**
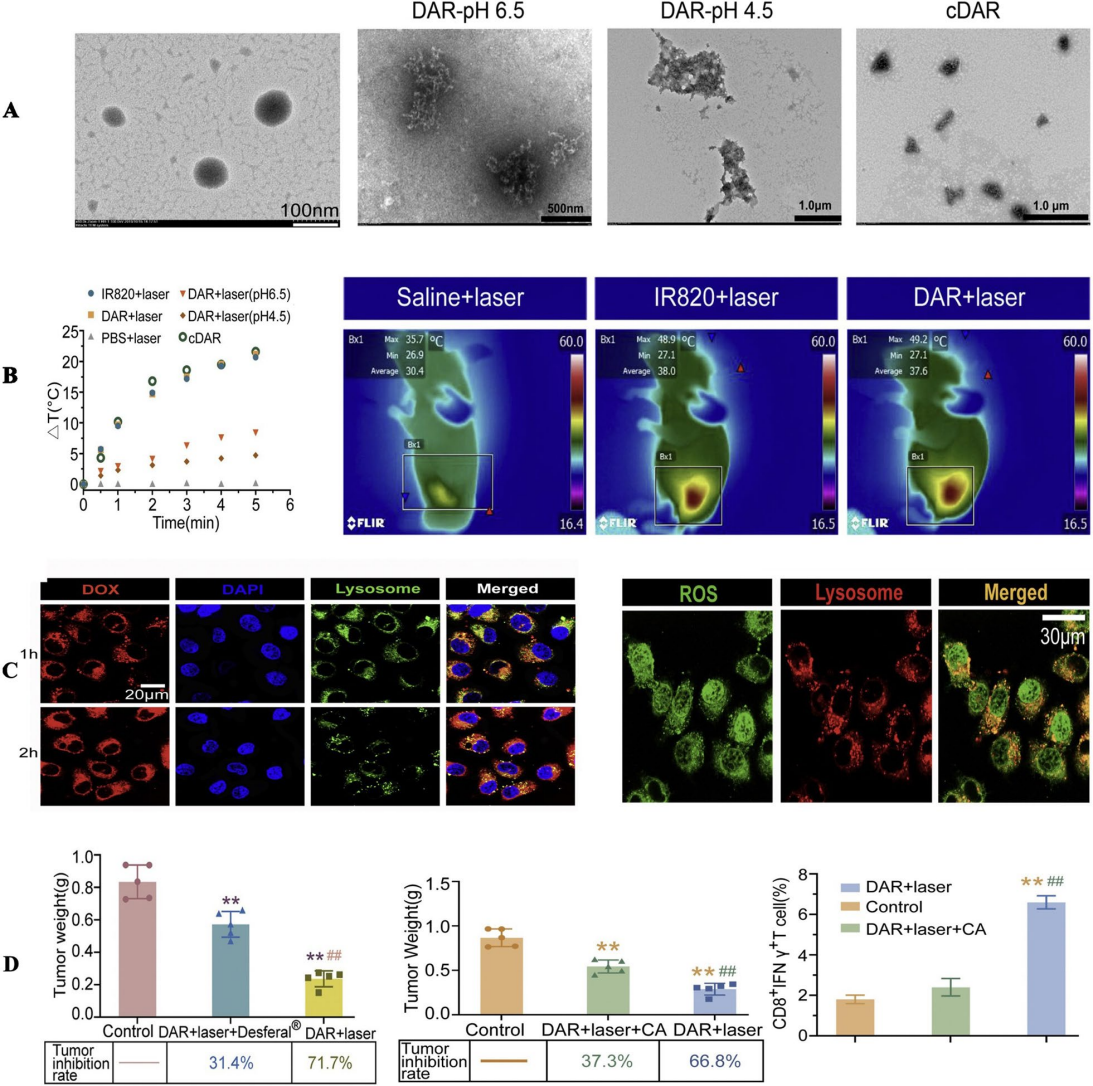
**A** The TEM image of DAR and its pH-triggered structure transition **B** The temperature elevation of DAR, PBS, IR820 and cDAR after laser irradiation. (left) and Infrared thermographic images of mice injected with saline, IR820 and DAR were tested at 5 min after laser irradiation (right). **C** The lysosome escape of DAR in MCF7 cells. Scale bar was 20 μm(left) and the lysosome distribution of ROS that produced DAR + laser treatment. Scale bar was 30 μm (right). **D** The MCF7(left) / 4 T1(right) tumor weight and tumor inhibition rate of different groups (n = 5). ⁎⁎p < 0.01 vs. control, ##p < 0.01 vs. DAR + laser + Desferal®; The percent of CD8 + IFNγ + T cells in tumor after different treatment (n = 3). ⁎⁎p < 0.01 vs. control, ##p < 0.01 vs. DAR + laser + CA. Copyright 2021, ELSEVIER publishing group

Iron-based nanoparticles have demonstrated powerful therapeutic effects in ferroptosis-mediated cancer therapy, as they are able to achieve targeted delivery of exogenous iron into tumor cells, catalyzing the Fenton reaction and triggering ferroptosis process. [208–210]. However, the biomedical applications of these nanomaterials may be limited due to the cytotoxic effects of high doses of iron [211, 212]. To address this issue, Xiong et al. [213] have designed an iron-free nano-activator (DAR) loading doxorubicin (DOX), tannic-acid (TA) and IR820 (Fig. 5), which could hijack intracellular iron to the Fenton reactions. They made best use of intracellular iron stockpiled in endogenous lysosome by means of using ROS-producers to increase the permeabilization of lysosomal membrane, triggering ferroptotic cell death and relevant oxidative stress by an intracellular positive feedback loop. This method provides a novel perspective on ferroptosis-based cancer immunotherapy through the effective use of endogenous iron. Employing ROS generators to trigger lysosomal membrane permeabilization or introducing swelling-type nanoparticles into lysosomes for inducing lysosomal membrane rupture has demonstrated effectiveness in releasing endogenous iron. This approach offers a solution that circumvents the toxicity concerns linked to exogenous iron when employing ferroptosis therapy. Effectively releasing intracellular iron holds substantial potential for advancing ferroptosis therapy. In addition to stimulating endogenous iron release, nanoparticles can also be able to deliver exogenous iron into tumor cells. For example, Zhang et al. [214] developed a self-amplifying nanodrug (RCH NPs), which consisted of hemin (ferric porphyrin), celecoxib and roscovitine. In this study, hemin was capable of converting intracellular hydrogen peroxide into toxic hydroxyl radicals via Fenton reaction. Particularly, both inflammation-related immunosuppression and IFN-g-associated adaptive immune resistance were eliminated due to the combined effect of the two drugs in the nanoparticles, strongly addressing the potential negative effects of ferroptosis in tumor immunotherapy. The RCH NPs showed brilliant biocompatible property and improved immunotherapy efficacy. Similarly, Han et al. [215] developed a Zn-pyrophosphate core–shell nanoplatform (ZnP@DHA/Pyro-Fe) to co-deliver Chol-DHA (Dihydroartemisinin) and Pyro-Fe to colorectal tumors in mouse models. Their results showed that reduced Pyro-Fe could catalyze the breakdown of DHA and then generate ROS in an O [2]-independent way, leading to ferroptotic tumor cells death. These researches impyed the potential of utilizing nanotechnology to repurpose DHA and other drugs with brilliant safety, including inducing increased ROS generation and causing notable tumor inhibition by co-delivering exogenous iron complexes.

Iron-free nanoparticles not only could induce ferroptosis via Fenton's reactions or Fenton-like reactions but also could be used to downregulate directly the intracellular level of glutathione (GSH) or glutathione-dependent peroxidases 4 (GPX4), causing the increasement of lipid peroxidation. For example, Li et al. [216] constructed glycyrrhetinic acid-based nanoparticles (GCMNPs), which could trigger ferroptosis through inhibiting GPX4 production. Moreover, in combination with ferumoxytol, GCMNPs augmented Fe-dependent cytotoxicity via the Fenton reaction. Cao et al. [216] also constructed gold nanoclusters (GNPIPP12MA) containing the FTO inhibitors, which were capable of selectively depleting GSH in AML cells and inducing tumor cells ferroptosis. In particular, GSH could mediate MA discharge and FTO inhibition, resulting in hypomethylation of target RNA transcripts and tumor cells reduction. Besides, song et al. [217] have developed intracellular-acidity-activatable dynamic nanoparticles (BNP@R) for tumor-targeting transportation of the ferroptosis inducer RSL-3, a GPX4 inhibitor. These nanoparticles could achieve acid-activatable PDT through protonation of the ionizable core, and efficiently increase tumor infiltration of T cells to secrete IFNγ, thereby sensitizing the cancer cells to ferroptosis induced by RSL-3. It can be seen that GSH plays a crucial role in protecting tumor cells from apoptosis by scavenging ROS. Depletion of GSH and subsequent inactivation of GPX4 result in excessive membrane lipid peroxidation, ultimately triggering ferroptosis. While GSH depletion and biosynthesis inhibition are desirable strategies for anti-cancer treatment, the precise biological mechanisms and potential side effects are not fully understood. Additionally, the short half-life of GSH and the off-target effects of GSH depletion in normal tissues limit therapeutic efficacy [218, 219]. Thus, there is an urgent need for a GSH/ GPX4-depleting system that offers high specificity, low toxicity, and can synergize with other cancer therapies.

### The application of nanoparticles-based pyroptosis in cancer therapy

As a form of regulated cell death (RCD), pyroptosis can exert the pro-inflammatory effect [220, 221], which is mainly attributed to the formation of transmembrane pores on tumor cells, and pyroptotic cell death occurs when gasdermin E (GSDME) or gasdermin D (GSDMD) is cleaved [78, 220]. Subsequently, the further lysis of cell membrane results in the discharge of inflammatory cytokines, such as IL-18, NK-κB, IL-1β, and so on, leading to activation of the immune response [222, 223]. Chemotherapeutic drugs are usually used to induce pyroptosis, whereas, they have high dependence on GSDMD level, thus the primary problem of pyroptosis induction is changing the state of GSDMD [224].

#### PDT/PTT-induced pyroptosis

**Fig. 6:**
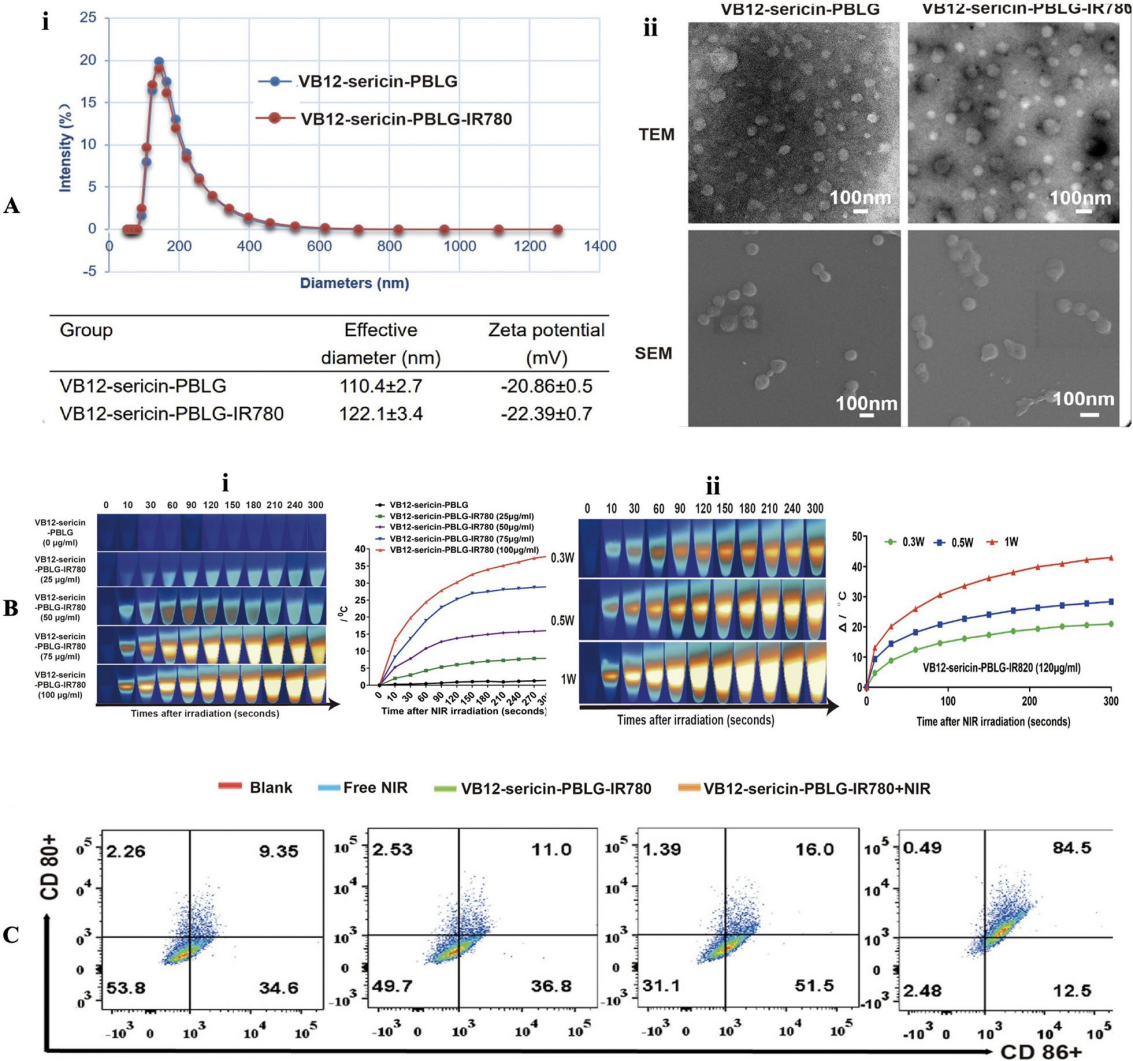
**A** i: Dynamic light scattering (DLS) results (top) and the corresponding size distributions and surface potentials of VB12-sericin-PBLG nanomicelles (bottom). ii: TEM and SEM images of micelles. Data are the mean ± SD, n = 5. **B** i: Thermal images (left) and temperature increase curve (right) induced by PBS, free IR780, and different concentrations of VB12-sericin-PBLG-IR780 nanomicelles under NIR irradiation (0.5 W/cm^2^, 5 min). ii: Thermal images (left) and temperature increase curve (right) induced by VB12-sericin-PBLG-IR780 nanomicelles (100 μg/mL IR780) under different powers of NIR irradiation (0.3, 0.5, and 1.0 W/cm^2^, 5 min). **C** Flow cytometry of DC maturation after treatment with HMON@IR820/Pt-NPs with or without NIR irradiation (0.7 W/cm^2^, 5 min). Copyright 2022, American Chemical Society publishing group

In the present, photothermal therapy (PTT) and photodynamic therapy (PDT), have been broadly applied for tumor therapy due to its brilliant accuracy, efficiency, and flexibility. Recently published studies have suggested that it is a feasible therapy measure to induce pyroptosis though photoactivation for tumor cell ablation. For example, Guo et al. [225] developed a novel IR780 loaded nanoparticle (VB12-sericin-PBLG-IR780) (Fig. 6), which could achieve highly efficient photothermal conversion (∼40%) and generating reactive oxygen species (ROS) simultaneously. Their results demonstrated that the combined effects of PDT and PTT can cause mitochondrial damage through downregulating ATP5MC3. As a result, Ox-mitoDNA generated and then increased the intracellular level of NLRP3 inflammasomes, Caspase-1 proteins and GSDMD, as well as promoted IL-1β and IL-18 release, leading to the activation of NLRP3/Caspase-1/GSDMD signaling pathway and pyroptotic tumor cells death, as well as the maturation of dendritic cells and the recruitment of CD4^+^ and CD8^+^ T cell. These nanomicelles may suggest a new PTT/PDT-mediated tumor therapy via mitoDNA oxidative damages, which improved cancer immunotherapy. Traditionally, pyroptosis can typically be triggered by chemotherapeutic drugs, but it heavily relies on the GSDMD or GSDME level. Therefore, altering the GSDMD/GSDME state to induce pyroptosis is a key consideration [226]. In recent years, PDT and PTT have gained widespread use in cancer treatment due to their exceptional precision, efficiency, and flexibility [227]. Similarly, these researches have shown that a photoactivated pyroptosis-based intervention strategy is a promising approach for ablating cancer cells.

PDT can also be combined with chemotherapy or immunotherapy. Xiao [228] et al. have constructed an innovative TME ROS/GSH dual-responsive nanoplatform (MCPP NPs). In this study, MCPP NPs co-delivered the cytotoxic agent PTX and phototoxic agent P18 to induce pyroptosis. Under laser irradiation, it was realized to control P18 release by generating ROS. Their results showed that DAMPs release caused by tumor cells pyroptosis could enhance DC maturation, activate T-cell proliferation, boost the efficacy of adaptive antitumor immune response and anti-PD-1. Similarly, Qiu and Su et al. [229] developed a pH-responsive nanoparticle loaded with immune modulators RGX-104 and classic photosensitizers Ce6 (MRC NPs). RGX-104 can remodel immunosuppressive TME by activating the transcription of ApoE and regressing the activity of myeloid-derived suppressor cells (MDSCs). The combined therapy of RGX-104 and PDT efficiently invoked GSDME-mediated pyroptosis and comprehensive immune responses, which proved to be an ingenious tactic to develop pyroptosis-induced immune boosters. Capitalizing on the benefits of noninvasive and controllable treatment, PDT has the potential to enhance immunogenicity, rendering tumors more responsive to immunotherapy. Recent research has increasingly focused on the synergistic effects of combining immunomodulators and photosensitizers for comprehensive tumor immunotherapy. Immuno-photo combination therapy has the capacity to transform an immunosuppressive TME into an immunogenic TME, amplifying the tumor's response to immunotherapy. While chemotherapy and phototherapy have both been shown to induce pyroptosis, traditional chemotherapeutic agents can trigger pyroptosis in normal cells due to latent drug resistance and unavoidable toxic effects. Additionally, photosensitizers without targeting mechanisms may distribute in normal tissues. Therefore, the development of TME-responsive nanodrugs is crucial to achieving tumor-specific therapy and reducing systemic toxicity. The mentioned researches exhibited remarkable immunomodulation and PDT performance, paving the way for enhanced pyroptosis-potentiated immunotherapy in cancer treatment.

#### Osmotic pressure-induced pyroptosis

In recent years, antitumor therapy strategies based on the production of reactive oxygen species have showed enormous promise in the medical field, including photodynamic therapy and chemodynamic therapy [230–232]. But the unsatisfactory ROS generation efficiency takes the blame for their low therapy efficacy due to the relative absence of O_2_ and H_2_O_2_ in the TME, confined light penetration depth, and so on [233, 234]. Thus, it is needed urgently to develop new agents capable of efficiently eliciting ROS production.

**Fig. 7:**
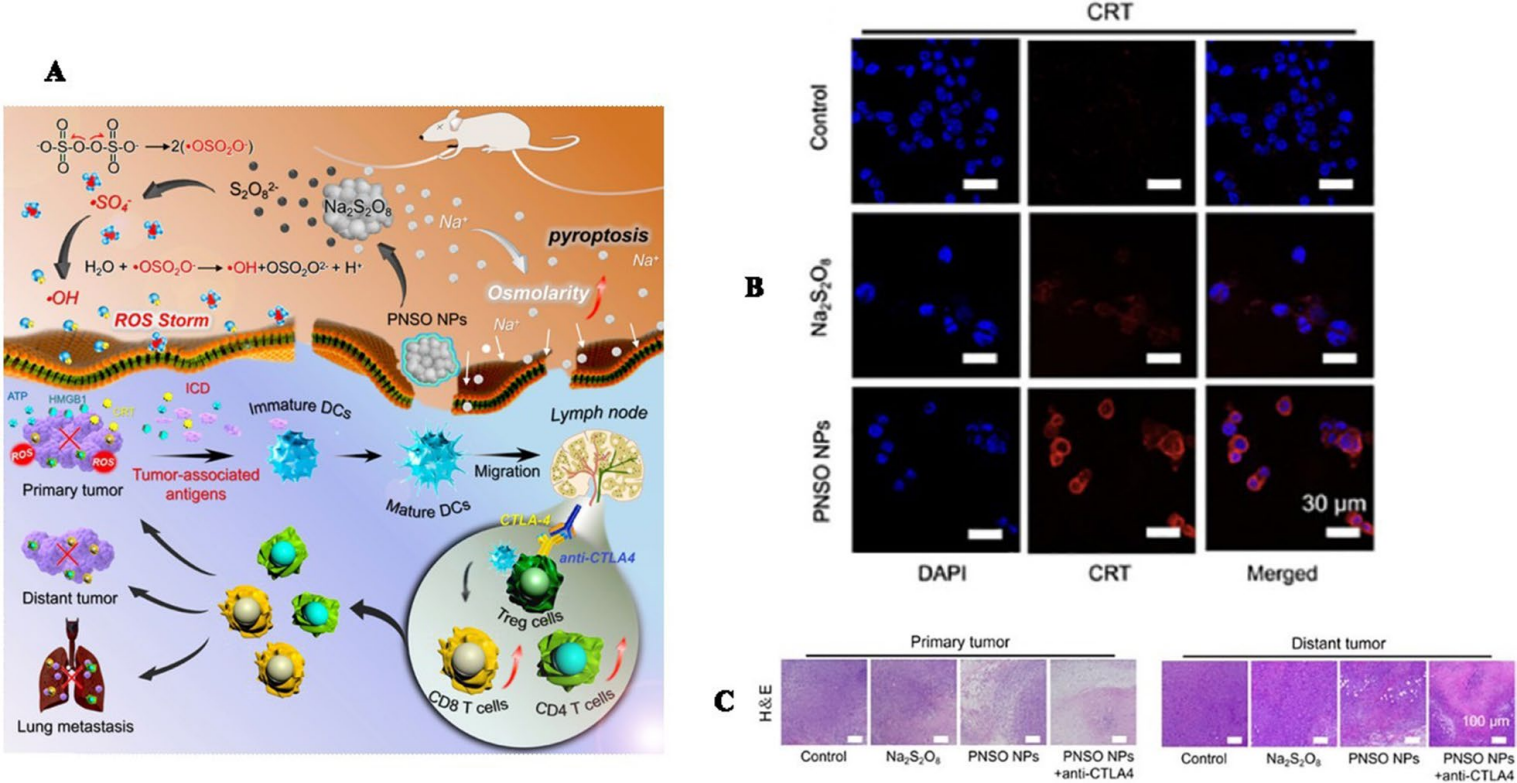
**A** Illustration of Therapeutic Mechanism of PNSO NPs **B** Detection of CRT exposure after 4T1 cells treated with PNSO NPs. **C** H&E staining images of tumor slides. Copyright 2020, American Chemical Society publishing group

Considering this situation, Liu et al. [235] have developed peroxydisulfate nanoparticles (sodium persulfate, Na_2_S_2_O_8_) as new ROS production agents (PNSO NPs) for in situ producing Na^+^ and S_2_O8_2_^−^ by stepwise degradation (Fig. 7). Benefiting from the ability of bypassing the cellular ion transportation rules via endocytosis, PNSO NPs can bring a lot of Na^+^ into the tumor cells, causing osmotic pressure surge and fast cell swelling and rupture. Their study indicated that these nanoparticles not only can efficiently kill tumor cells by the combined effect of surge of osmolarity and ROS but also can activate caspase-1-dependent pyroptosis, eventually triggering overall immune responses. Similarly, Ding et al. [236] reported biodegradable nanoparticles K_3_ZrF_7_: Yb/Er UCNPs (ZrNPs) to induce pyroptosis, which could trigger intracellular osmotic pressure surge by discharging large numbers of ions, leading to ROS increasement and caspase-1 protein activation, ultimately cell capture and lysis. They also confirmed that ZrNPs could induce strong tumor cells pyroptosis with excellent immunostimulatory activity proved by the augmented DCs maturity and the increased number of tumor-specific T cells, along with markedly inhibited tumor growth and metastasis. It's a well-known fact that pyroptosis is typically induced by chemotherapeutic drugs, which limits its broader applications due to issues like drug resistance and severe side effects [237]. Hence, there is a critical and pressing need for the exploration and development of pyroptosis activators. Research has shown that surpassing the threshold of ROS levels in tumor cells can lead to oxidative damage in cellular components, potentially resulting in cell apoptosis or necrosis [238]. Additionally, ROS-mediated dynamic therapy has been found to be highly immunogenic, as it can trigger acute local inflammation [239]. These two studies suggested that the abrupt increase in ions within cells raised intracellular osmolarity and disrupted homeostasis, which, in turn, leaded to elevated oxidative stress and an increase in ROS. This, in an orderly manner, activated the nucleotide-binding oligomerization domain-like receptor protein 3 (NLRP3) inflammasome and caspase-1 proteins, resulting in GSDMD cleavage and IL-1β maturation. Therefore, cancer therapeutic strategies based on ROS-induced pyroptosis hold significant promise in the medical field.

The increase of osmotic pressure in the organelles can also induce pyroptosis process besides occurrence in the cytoplasm. Zheng et al. [240] have deigned Biodegradable Ca^2+^ nanomodulators (CaNMs) as pyroptosis inducers. The nanomodulators could trigger an abrupt surge in mitochondrial Ca^2+^ ions and rapidly cause mitochondrial Ca^2+^ overload, contributing to ROS increasement, cytochrome C secretion, caspase-3 protein activation, GSDME cleavage, and eventually the tumor cells pyroptosis. Their results similarly revealed the strong immune responses mediated by CaNMs, observably inhibiting tumor proliferation and lung metastasis. They showed that mitochondrial Ca^2+^ overload has the pyroptosis-inducing capability. It has been reported that specific ions [241], molecules, or chemotherapeutic drugs could trigger GSDMD or GSDME-mediated pyroptosis under certain conditions [242, 243], but these small molecules are still plagued by systemic effects related to fast blood flow, non-specific biodistribution, and unfavorable responses. Mitochondria, as essential organelles in cells, play a crucial role in cell growth by maintaining a dynamic balance between free Ca^2+^ and bound Ca^2+^ [244]. When this balance is disrupted, cytochrome C is released from mitochondria, activating caspase-3 and leading to apoptosis, as well as pyroptosis [245]. The Ca^2+^ nanomodulators have proven effective in suppressing tumor proliferation through the mitochondrial Ca^2+^ overload-mediated apoptosis pathway or the ICD pathway [246, 247]. Therefore, it is prudent to explore whether Ca^2+^ nanomodulators could be better employed in cancer treatment through the pyroptosis pathway.

#### Other types of pyroptosis-induced nanoparticles

**Fig. 8:**
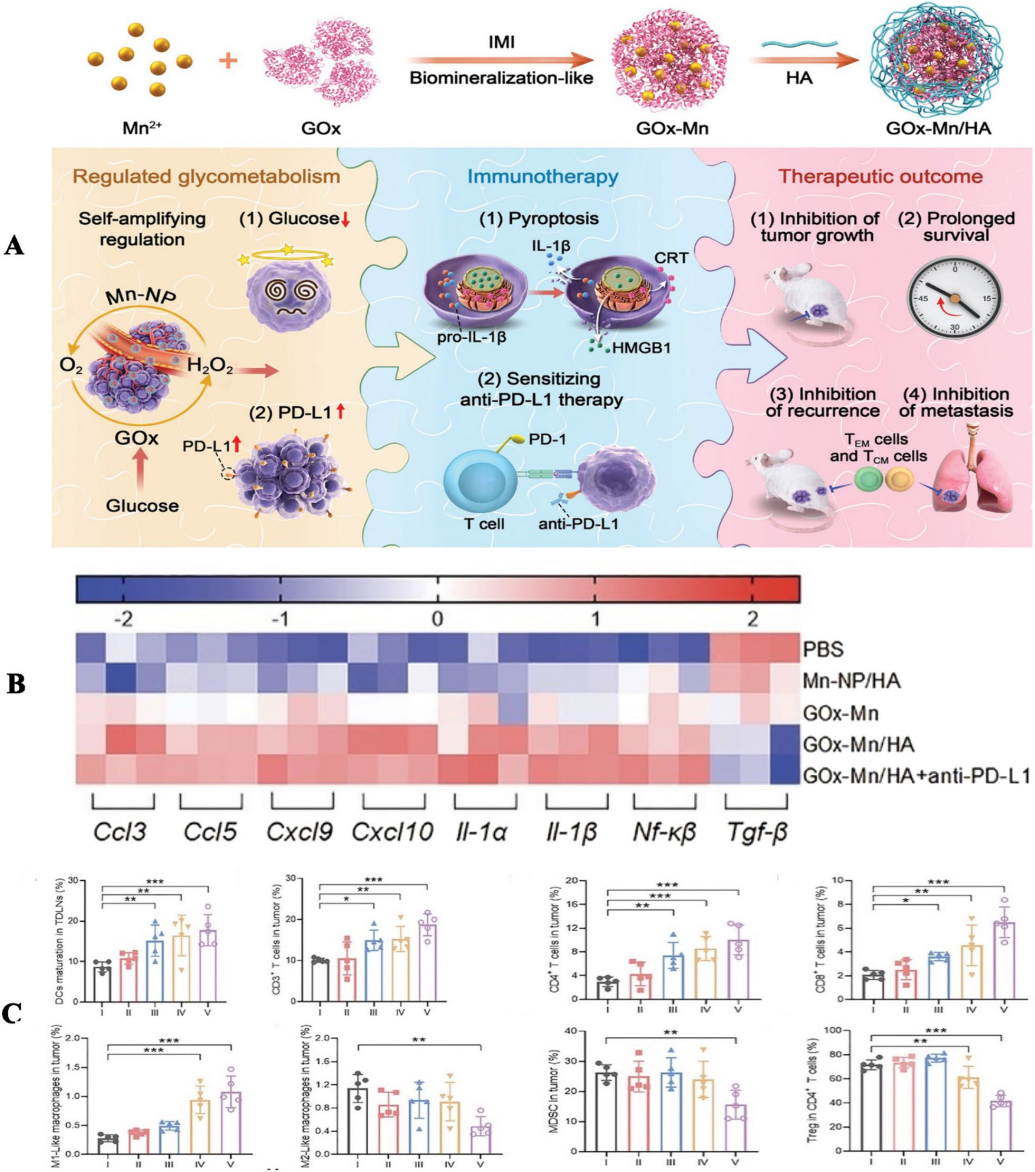
**A** Scheme of GOx-Mn/HA synthesis and biomineralized two-enzyme nanoparticles that regulate tumor glycometabolism inducing tumor cell pyroptosis and robust antitumor immunotherapy. **B** Multiple gene expression levels in tumors characterized by RT-PCR. **C** Tumor immune microenvironment (TIME) reprogramming. Ratios of DCs maturation in TDLNs; Ratios of CD3 + T cells in tumors; Ratios of CD4 + T cells in tumors; Ratios of CD8 + T cells in tumors; Ratios of M1-like macrophage in tumors; Ratios of M2-like macrophage in tumors; Ratios of MDSCs in tumors; Ratios of Treg cell in CD4 + T cells. Copyright 2022, WILEY publishing group

Currently, to improve immunotherapy's therapeutic efficacy, it is a useful method to combine immune checkpoint treatment with chemoradiotherapy. However, chemotherapy and radiotherapy usually bring about severe side effects, so exploring a more safe and effective strategy is undoubtedly vital. Studies have indicated that controlling cell metabolism is also an effective therapeutic strategy in cancer treatment while the major modality of tumor glycometabolism is glycolysis [248, 249]. For example, Zhang et al. [250] have reported that modulating cancer cells glycometabolism could promote pyroptotic cell death. They constructed double-enzyme GOx-Mn nanoparticles (Fig. 8), which integrated glucose oxidase (GOx) and Mn-containing nanozymes to achieve continuous amplification of glucose consumption. They further combined these nanoparticles and ICBs to treat 4T1tumor-bearing mice, the inhibition rate of which arrived at 92.9%, together with the greatly extended mice's survival time. Thus, their study indicated that regulating tumor glycometabolism could be a potential strategy combined with immune checkpoint therapy for efficient cancer immunotherapy. Moreover, the regulation of tumor metabolism has advantages of fewer side effects and more brilliant therapy efficacy compared with chemotherapy or radiotherapy. It is also remarkable to discover that depleting glucose from tumor cells could induce pyroptosis and stimulate a robust tumor immune response. Furthermore, this study has shown that glucose depletion led to increased expression of PD-L1 in tumor cells, thus enhancing the efficacy of ICBs treatment targeting the PD-L1/PD-1 pathway. These outcomes deserve researchers’ further exploration.

**Fig. 9:**
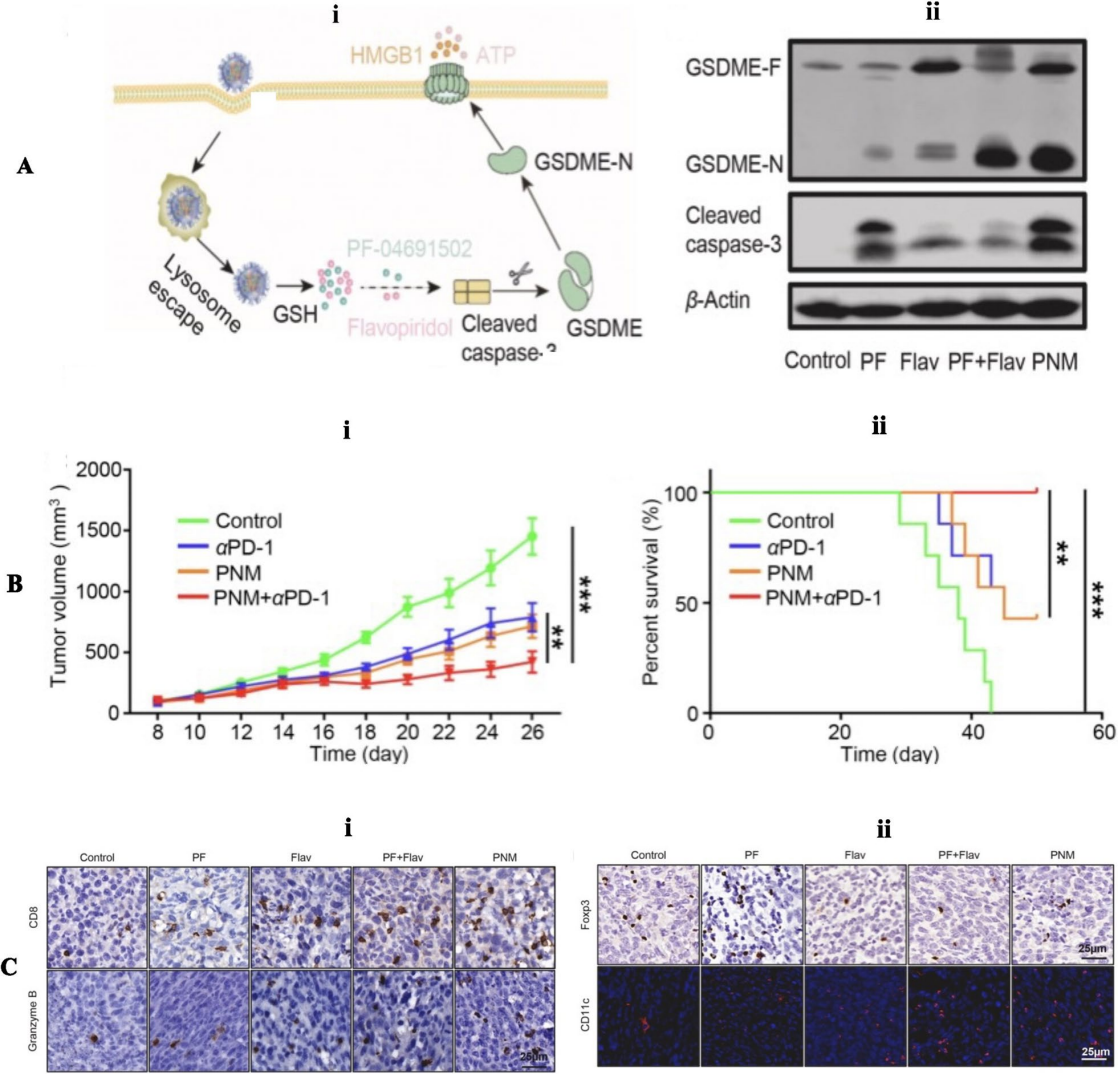
**A** i: Mechanistic illustration of PNM-induced pyroptosis in tumor cells. ii: GSDME-N and cleaved caspase-3 protein expression in different groups of tumor tissues by Western blotting. **B** Improved antitumor effect of PNM in combination with αPD-1. i: The tumor growth curve of mice in different treatment groups (n = seven mice per group). ii: The survival curve of mice in different treatment groups (n = seven mice per group). **C** i: Representative immunohistochemical images of CD8 and immunofluorescence of Granzyme B in tumor sections in different groups (scale bar = 25 μm). Data are shown as the mean ± SEM (*P < 0.05; ***P < 0.001). ii: Representative immunohistochemical images of Foxp3 and immunofluorescence of CD11c in tumor sections in different groups (scale bar = 25 μm). Data are shown as the mean ± SEM (*P < 0.05;***P < 0.001). Copyright 2022, Elsevier publishing group

It has been confirmed that oncogenic signaling plays a vital role in tumor immune evasion [251]. The activation of PI3K, one of the mutated genes in many solid tumors [252, 253], could trigger the PI3K/Akt/mTOR signaling pathway, inhibiting functional performance of cytotoxic T cells and decreasing tumor infiltration of immune cells, eventually causing resistance to T cells-mediated immunotherapy [254]. Therefore, Yang et al. [255] developed a prodrug nanomicelle (PNM) activated by TME, which co-delivered the PF-04691502 (PF) and flavopiridol (Flav), respectively as the PI3K/mTOR inhibitor and the broad spectrum CDK inhibitor (Fig. 9). They reported that PNM successfully induced GSDME-dependent immunogenic pyroptotic cell death and boosted tumor cells immunogenicity, contributing to DC maturation. Activation of the traditional apoptotic marker cleaved caspase-3 can serve as a regulator of pyroptosis, selectively cleaving GSDME to shift apoptosis into pyroptosis. This process relys on the intracellular levels of GSDME expression [73]. Previous studies have suggested that molecular inhibitors targeting oncogenic signaling pathways can also induce GSDME-mediated pyroptotic death in tumor cells [256, 257]. In this study, small molecule-targeted drugs were used to simultaneously inhibit PI3K/mTOR and CDK, triggering pyroptosis in cancer cells and thereby enhancing the effectiveness of immune therapy. However, it's worth noting that the activation of PI3K/mTOR and CDK signaling pathways is also observed in normal tissues, which may cause adverse effects and reduce the bioavailability of molecular inhibitors [258, 259]. Additionally, healthy cells are susceptible to cytotoxic agent-induced pyroptosis due to the extensive expression of gasdermins in normal tissues [224]. Therefore, the challenge lies in improving the tumor-targeting ability and minimizing the systemic toxicity of small molecular inhibitors, which is crucial for their clinical application [260]. In this research, stimuli-responsive prodrug nanomedicines played a vital role as a drug delivery system, realizing tumor-specific therapy and reducing systemic toxicity by minimizing the exposure of healthy tissues to cytotoxic agents.

**Fig. 10:**
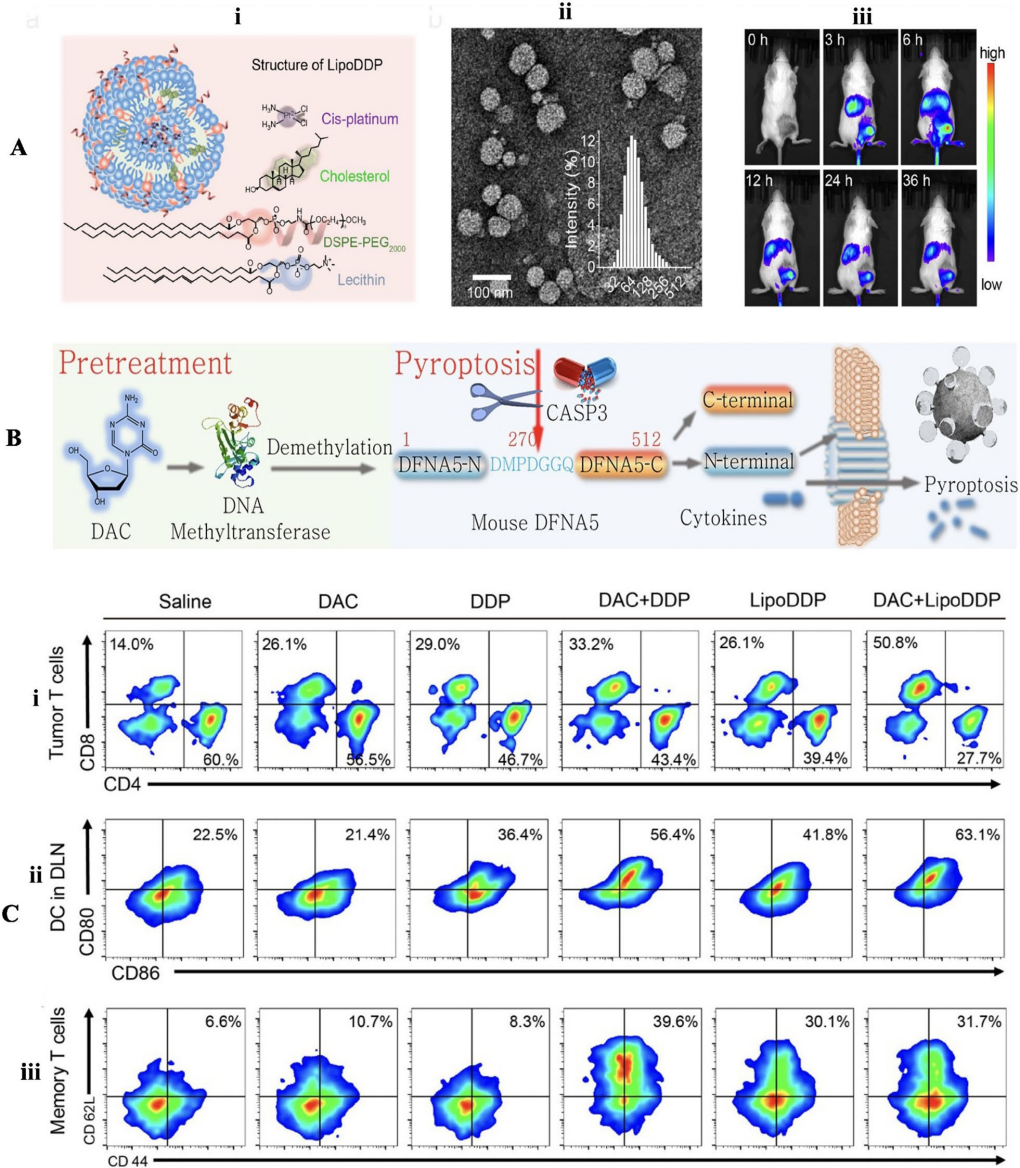
**A** i: Schematic representation of LipoDDP synthesis ii: TEM image and hydrodynamic size distribution of LipoDDP (scale bar: 100 nm). iii: Optical living imaging of 4 T-1 tumor-bearing mice after treated with Dir-labeled LipoDDP at different time periods. **B** Illustrative diagram of tumor cell pyroptosis triggered by DAC/chemotherapeutics **C** Pyroptosis of tumor cells improved immune response of chemotherapy. i: Representative flow cytometric analysis of CD8 + and CD4 + T cells gating on CD3 + cells in the tumors. ii: Representative flow cytometric analysis of CD80 + CD86 + cells gating on CD11c + cells within TDLN. iii: Representative flow cytometric analysis of CD44 + CD62L + cells gating on CD8 + cells within the spleen. Copyright 2019, American Chemical Society publishing group

In the present, most chemotherapy drugs kill tumor cells by activating caspase-3-mediated apoptosis, which indicates chemotherapy can also induce pyroptosis mediated by GSDME and antitumor immune responses of itself [261]. Unfortunately, on account of the promoter methylation of DFNA5 gene, most of mouse cancer cells may express much less GSDME than other normal cells [262, 263]. Here, Fan et al. [237] proposed an approach of incorporating decitabine (DAC) with chemotherapy nano-agents to induce pyroptotic tumor cells death by epigenetics (Fig. 10). They pre-performed DAC with experimental mice for the DFNA5 gene demethylation in cancer cells and subsequently administrated drugs using a common tumor-targeted nano-liposome containing cisplatin (LipoDDP). Their experiments demonstrated that GSDME silencing in tumor cells was reversed, caspase-3 pathway was activated and pyroptosis was induced. Alternatively, apart from promoting the intracellular expression of GSDMs, they could also be delivered directly into tumor cells. Wang and colleagues [77] have developed a biorthogonal system, which can convey GSDMA3 into tumor cells and control its discharge. They constructed an ortho-carbamoylmethylene silyl-phenolic ether system as a carrier to deliver GSDMA3. Their results showed the augmented tumor infiltration of tumor-specific T cells and NK cells, accompanied by the downregulation of CD4^+^FOXP3^+^ T regulatory cells (Tregs) level. Moreover, the number of immunostimulatory M1 macrophages increased whereas the M2 macrophages reduced. They have investigated a combination therapy involving DNA demethylation and chemotherapy to induce pyroptosis in tumor cells. This combined approach generated a robust immune response, thereby providing a new inspiration for designing novel pyroptosis-induced nanomedicines in cancer treatment.

These studies have indicated that the high intracellular level of GSDMs plays a vital role in increasing the antitumor immunostimulatory ability of dying tumor cells, which could be achieved by means of nanoparticles, as a safe and efficient approach.

### The application of nanoparticles-based autophagy in cancer therapy

#### Autophagy-induced nanoparticles

**Fig. 11:**
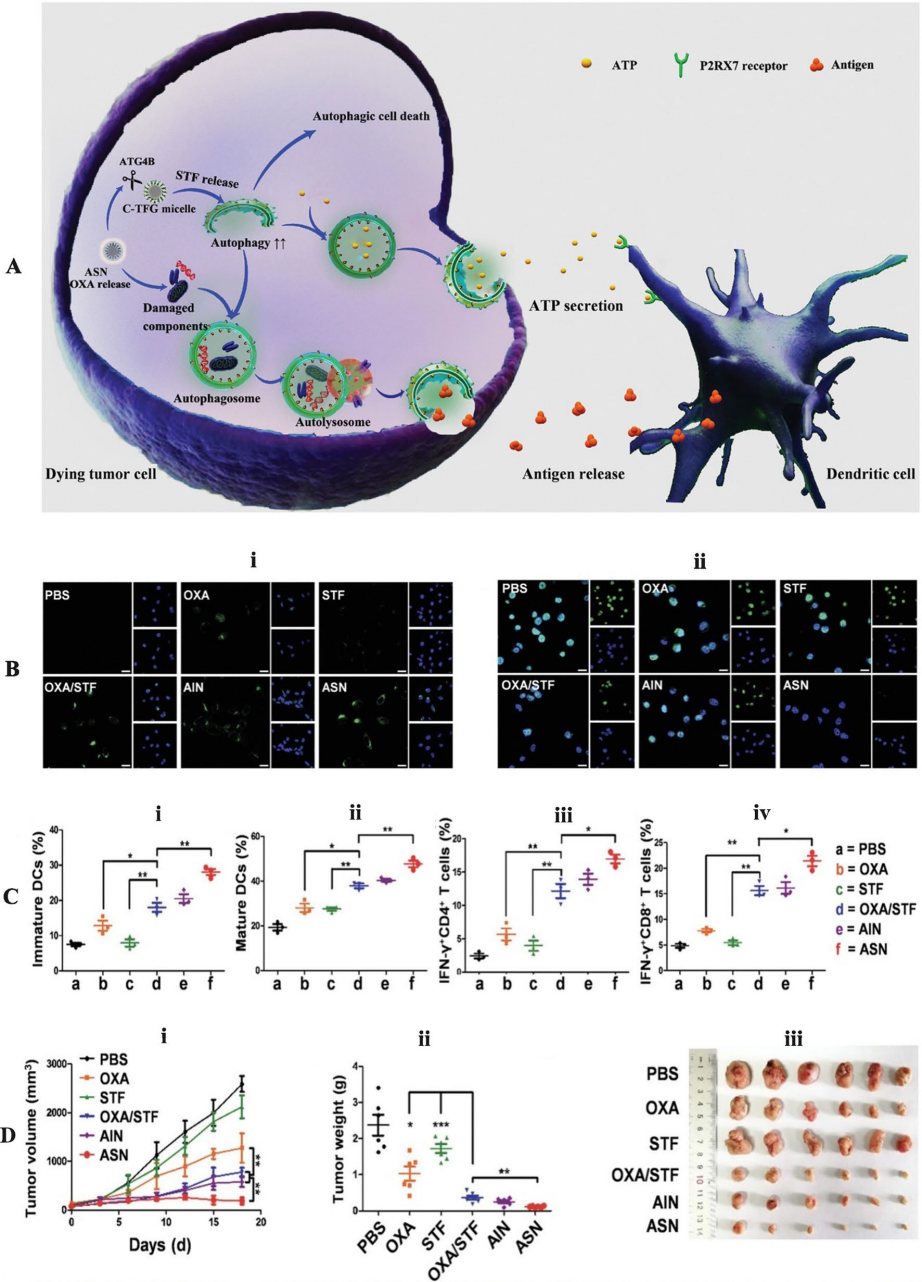
**A** Simplified mechanism of OXA-induced immunogenic cell death and autophagy-mediated DCs recruitment. **B** i: CLSM examination of CRT exposure in CT26 cells (scale bars: 10 µm) ii: CLSM examination of HMGB1 location in CT26 cells (scale bars: 10 µm) **C** ASN enhanced immune stimulation in vivo. i: Frequency of immature DCs in tumor tissue (n = 3). ii: Frequency of mature DCs in spleen (gated on CD11c + cells, n = 3). iii: Intratumoral infiltration of IFN-γ + CD4 + T cells post-treatment (gated on CD3 + , n = 3). iv: Intratumoral infiltration of IFN-γ + CD8 + T cells post-treatment (gated on CD3 + , n = 3). **D** ASN enhanced antitumor efficiency in vivo. i: Tumor growth curve during treatment (n = 6). ii: Tumor weight of CT26-bearing mice after treatment (n = 6). iii: Representative tumor images of mice post-treatment. Copyright 2020, WILEY publishing group

Antitumor immune responses based on chemoimmunotherapy highly depend on tumor cells autophagy, which plays a vital role in cancer immunotherapy [96]. When cancer cells are under chemoimmunotherapy, excessive autophagy activation can cause more cancer cells death and promote the tumor-associated antigens presentation and inflammatory factors release of dying cells [65]. Nonetheless, it is still a difficult matter to timely and precisely hyperactivate tumor autophagy during chemoimmunotherapy. To solve this issue, Wang et al. [264] constructed an autophagy-based drug delivery system (ASN) loaded with autophagy inducer STF-62247, and oxaliplatin prodrug (HA-OXA) (Fig. 11). In their study, when ASN entering cancer cells, the HA-OXA shell of ASN firstly responded to the reductive TME and then released oxaliplatin to induce ICD, mildly triggering autophagy process. In response of the “mildly activated” autophagy level, the C-TFG micelles could rapidly release STF-62247, a powerful autophagy inducer, further achieving the transformation of “mildly activated” state to “excessively activated” state. Their results showed that ASN could induce a brilliant antitumor immune response and demonstrated an excellent tumor growth suppression effect in CT26 tumor. Similarly, Ge et al. [265] established a pH-responsive nano­carrier, CUR-BMS1166@ZIF-8@PEG-FA (CBZP), loaded with curcumin (CUR) and BMS1166 for autophagy induction and immune checkpoint treatment. The study demonstrated that combined treatment of BMS1166-mediated ICB immunotherapy and nanodrugs-based autophagy activated a strong antitumor immunity and remodeled the TIME in Osteosarcoma (OS), contributing to a satisfactory therapeutic efficacy and robust immunological memory. In general, chemotherapy drugs tend to stimulate autophagy, but often only to a "mildly activated" state rather than to an "over-activated" state. This mild activation can have a protective effect on tumor cells rather than inducing their death. In these studies, chemotherapy drugs were combined with interventions to "over-activate" autophagy, not only did more tumor cells undergo autophagic death, but it also enhanced antigen presentation from dying cells and increased the secretion of immune stimulators. This combination approach aimed to achieve optimal antitumor immunity. Therefore, the strategy of timely and accurately over-activating autophagy is a win–win approach. It eliminates the cytoprotective function of autophagy while simultaneously promoting immune stimulation in chemotherapy-treated tumor cells.

**Fig. 12:**
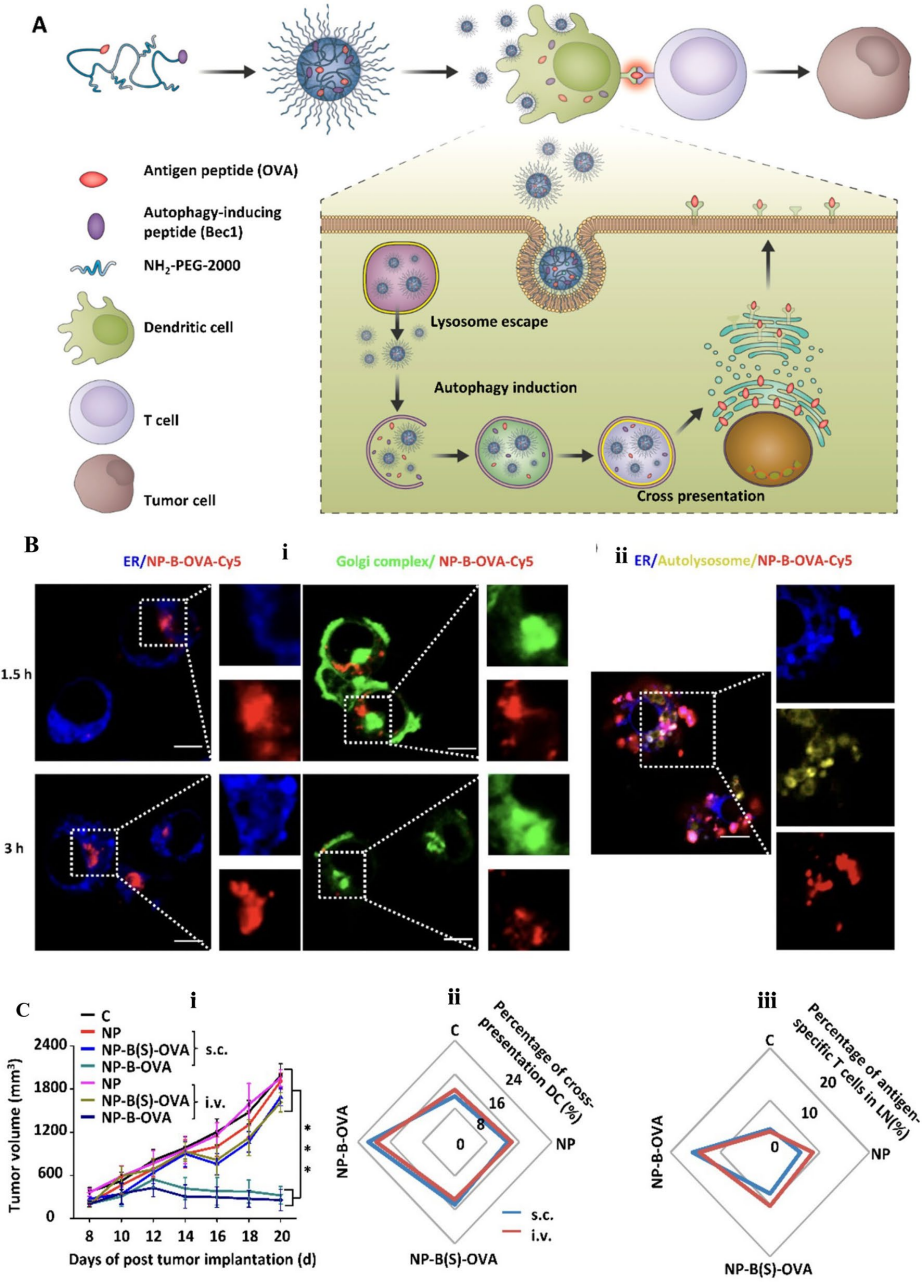
**A** Schematic of nanoactivtors inducing autophagy for cross-presentation and priming T cells. **B** Nanoactivator-mediated autophagic flux detection. i: Co-localization organelles ER and Golgi complex with NP-B-OVA-Cy5 after incubation for 1.5 h (upper panel) and 3 h (lower panel); white dashed boxes indicate the co-localization signals; blue, ER-tracker; green, Golgi complex; red, NP-B-OVA nanoparticles, n = 3. ii: Confocal images of DC2.4 cells 2 h post NP-B-OVA-Cy5 incubation and stained with indicated organelles probes. Scale bar, 5 μm, n = 3. **C** in vivo antitumor efficacy of nanoactivator. i: Tumor volume changes of B16-F10-OVA bearing mice with indicated treatments, n = 6***p < 0.001; one-way ANOVA for indicated comparison. ii: Percentages of cross-presentation DCs in lymph node 7 d after indicated treatments. iii: Percentages of antigen-specific CD8 + T cells isolated from the spleen 7 d after indicated treatments. Copyright 2019, American Chemical Society publishing group

Autophagy process can also play a basic role in antitumor immune response, such as TAAs presentation and tumor-specific T cells activation [266]. Li et al. [267] have devised a drug composite based on polyglycerol-functionalized nanodiamonds loaded with doxorubicin (Nano-DOX). It was confirmed to induce autophagy rather than apoptosis in glioblastoma cell (GC) and provoke GC to emit antigens and DAMPs, leading to maturation of DCs and reversal of immunosuppressive microenvironment in glioblastoma multiforme (GBM). Apart from inducing autophagy of tumor cells, nanomaterials have also been applied to induce autophagy of immune cells. For example, Wang et al. [268] developed an autophagy assisted method for DC-mediated immunotherapy by synthesizing self-assembled nanoparticles (NP-B-OVA) with autophagy-inducing ability (Fig. 12), which could be phagocytosed by DCs within 1 h and trigger autophagy to process antigens. Their results suggested that efficient antigen processing and presentation in bone-marrow derived dendritic cells (BMDCs) treated with nanoactivators increased obviously, compared with cells in the control groups that failed to activate autophagy. Hence, the strategic manipulation of the autophagy process presents a promising and effective approach to leverage the immune system's potential. Nanomaterials with precise physicochemical properties have demonstrated remarkable efficacy in modulating autophagy, delivering antigens, and enhancing immunotherapy. This approach holds the potential to serve as a potent tool for cancer immunotherapy with translational capabilities. Therefore, the well-thought-out design of nanomaterials for use in autophagy modulation strategies may provide multifunctional capabilities and spatiotemporal control over immune cells, ultimately enhancing anti-cancer activities while ensuring improved safety profiles.

#### Autophagy-inhibited nanoparticles

Aa a typical cytoprotective program, autophagy enables the clearance of damaged intracellular proteins or organelles and the evasion from external stimuli or stress, such as oxidative stress, mechanical harm, cytotoxic agents, and pathogen invasion [269]. However, autophagy process is not always immunogenic. In particular, autophagy is considered to be a double-edged sword in cancer progression [270]. In early tumorigenesis, it has the ability of tumor inhibition by relieving oxidative stress and genomic instability. By contrast, in established tumors, the autophagy activation can generate metabolic precursors, which are necessary for tumor cells to meet the increased metabolic requirement. This facilitates sustainable tumor cells growth even under nutrient deprivation conditions [271]. In addition, it is reported that autophagy could prevent cancer cells against hypoxia- and chemotherapy-elicited death [272]. Thus, suppressing autophagy is also recognized as a valid approach in tumor therapy [273].

**Fig. 13:**
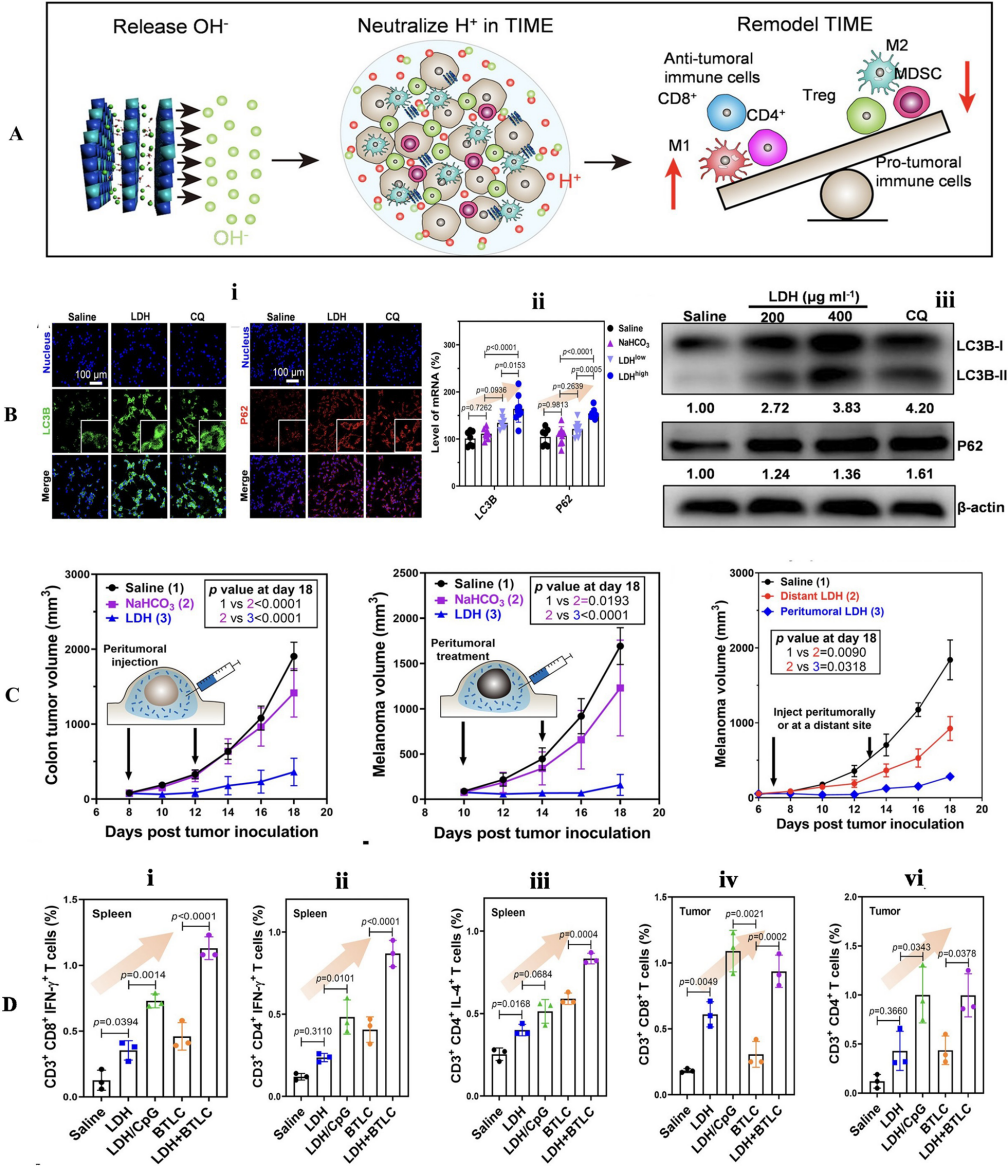
**A** Schematic illustration of the changes of immune cells in TIME with the treatment of LDH NPs. **B** LDH NPs inhibit tumor cell proliferation by interfering with autophagy. i: The expression of LC3B (left) and P62 (right) proteins in CT26 cells treated with LDH NPs (400 μg mL^–1^) or CQ (10 μM). The nucleus was stained by DAPI (blue). ii: The levels of LC3B and P62 mRNA in CT26 cells-treated with LDH NPs (low, 200 μg mL^–1^; high, 400 μg mL^–1^) for 24 h. iii: The levels of LC3B and P62 proteins in CT26 cells-treated with LDH NPs for 24 h were detected by Western blot. **C** Antitumor efficacy of LDH NPs against colon tumor and melanoma. **D** i: The level of Trp2-specific CD3 + CD8 + IFN-γ + T cells in splenocytes (n = 3). ii: The level of Trp2-specific CD3 + CD4 + IFN-γ + T cells in splenocytes (n = 3). iii: The level of Trp2-specific CD3 + CD4 + IL-4 + T cells in splenocytes (n = 3). iv: The level of CD3 + CD8 + effector T cells in tumor (n = 3). vi: The level of CD3 + CD4 + T helper cells in tumor (n = 3). Data are means ± SEM. Copyright 2022, American Chemical Society publishing group

Based on this viewpoint, Zhang et al. [274] designed weakly alkaline layered double hydroxide nanoparticles (LDH NPs) (Fig. 13), which could block the autophagy process mediated by lysosomes in cancer cells by neutralizing the excess acid. According to their study, the immunosuppressive TME comes into being mainly due to the aerobic glycolysis of cancer cells, this process causes an increase in the acidity of the tumor microenvironment, resulting in immune resistance of tumor cells to tumor-infiltrating T cells and conversion of M1 macrophages to M2 macrophages [275–277]. They demonstrated that this approach reduced the level of immunosuppressive M2 macrophages, Tregs, and increased the level of immunostimulatory cells such as M1 macrophages and cytotoxic T lymphocytes. Lysosomal exocytosis is a significant mechanism for expelling the surplus protons (H^+^) produced during aerobic glycolysis into the extracellular environment [278]. Hence, interfering with lysosomes in cancer cells presents an alternative approach to hinder the acidification of the tumor microenvironment. What’s more, lysosomes also hold essential functions in the autophagic process of cancer cells by facilitating the maturation of autophagosomes [279, 280]. As a result, treatments aimed at disrupting lysosomes deserve deeper exploration for potential use in cancer therapy.

Similarly, Chen et al. [281] developed a PTT-activated in situ self-assembly nanoparticle (DMN). In this study, DMNs could co-load IR780 and chloroquine (CQ) as photosensitizers and autophagy inhibitors. Under NIR light irradiation, the photothermal-motivated nanoparticle validly penetrated into the deeper tumor tissues, promoting the phototoxicity of IR780 and accurately ablating tumor cells through autophagy inhibition. Besides, as an immune modulator, CQ could also reset tumor-associated macrophage cells (TAMs) toward the M1-like by activating NF-κB. The DMN successfully remodeled immunosuppressive tumor microenvironment by regulating the polarization of tumor-associated macrophages and recruiting tumor-specific T cells. Photoimmunotherapy (PTI) holds great promise for treating localized malignancies. However, PTI's effectiveness has room for improvement because tumor cells can often develop reversible resistance to the therapeutic stress induced by PTI. This resistance occurs due to autophagy in tumor cells, an intrinsic self-protection mechanism, which helps remove damaged organelles resulting from PTI [282]. This research focused on inhibiting autophagy to weaken the self-protection mechanism in tumor cells, enhance the phototoxicity of the photosensitizer, and ultimately boost the antitumor immune response. Therefore, the combination of autophagy modulation, particularly autophagy inhibition, with phototherapy, may be a promising strategy in cancer therapy.

### Conclusion and Prospect

Tumor immunotherapy has emerged as a promising approach for the treatment of cancer, harnessing the body's immune system to target and destroy cancer cells. While immunotherapy has shown remarkable potential, it faces several challenges, including off-target effects and the immunosuppressive tumor microenvironment. Nanomedicine offers a unique set of tools and design principles to address these challenges and enhance the therapeutic efficacy of immunotherapy. Based on the discussion above Here, we elucidate the design concepts and treatment outcomes associated with nanomedicine in the context of tumor immunotherapy and immune stimulation.

1. Improving drug delivery efficiency: One of the design goals of nanomedicines is to improve the efficiency of drug delivery in the body. Tumor immunotherapy usually requires the use of biological agents, such as immune checkpoint inhibitors, cytokines, nucleic acids or antibodies. These substances activate or enhance the immune system's response, including the production of T-cells, B-cells and antibodies, but these drugs may be rapidly cleared in the body, diminishing their efficacy. Nanomedicines can increase the concentration of drugs in tumor tissues by improving their solubility and stability, protecting them from degradation and prolonging their circulation time in the body through suitable carriers such as lipid nanoparticles or polymer nanoparticles.

2.Targeting tumor tissues: Nanomedicine design also includes the ability to target tumor tissues. Surface modification of nanoparticles allows for specific targeting of tumor cells or immune cells, reducing off-target effects and improving drug delivery to the tumor site. This targeting helps to increase the local concentration of the drug in the tumor tissue and reduce the damage to healthy tissue. Nanomedicines can target immune cells such as antigen-presenting cells, dendritic cells and T cells. This helps to improve the interaction between antigen-presenting and immune cells, thereby promoting stronger immune stimulation.

3. Reduced side effects and Controlled release of drugs: Nanomedicines can reduce side effects by improving the biodistribution of the drug and reducing the impact on non-target organs, while enhancing the local concentration of the drug in immune tissues. Nanomedicines can also be designed to enable controlled release of drugs, prolonging their presence in tumor tissue. This helps to reduce the frequency of treatment and improves patient convenience.

4. Promoting immune response: Nanomedicines can also be designed to improve immune cell function. Some nanoparticles can be designed as appropriate delivery systems to direct antigens to antigen-presenting cells, thereby enhancing T-cell activation. In addition, some nanomedicines can carry immunomodulatory molecules, such as cytokines, to modulate the immune response and improve the efficacy of immunotherapy. Nanoparticles can be engineered to modulate the immunosuppressive tumor microenvironment, making it more conducive to immune cell activity. Some nanoparticles can trigger immune responses, acting as adjuvants to enhance the activation of immune cells, particularly antigen-presenting cells.

5.Achieving combination therapies: Nanoparticles can carry multiple agents, enabling combination therapies that target multiple facets of the immune response and the tumor microenvironment simultaneously.

Nanomedicines have broad application prospects in tumor immunotherapy and have received extensive attention from researchers. The rational design of nanomedicines can effectively trigger the programmed death mode of tumor cells These include apoptosis, programmed necrosis, ferroptosis, autophagy and pyroptosis. In terms of therapeutic effects, nanomedicines have the following advantages in tumor immunotherapy:

1. Improved Drug Pharmacokinetics: Nanomedicine provides better drug solubility and stability, leading to improved pharmacokinetics and prolonged circulation of immunotherapeutic agents in the bloodstream.
2. Enhanced Targeting and Minimized Side Effects: Surface functionalization allows for improved tumor-specific drug delivery, reducing systemic toxicity and enhancing the concentration of drugs at the tumor site. More importantly, the precise control over drug release and targeting reduces side effects and enhances the safety profile of immunotherapies. This makes treatments safer and more tolerable for patients.
3. Enhanced Immune Activation and Reduced Immunotherapy Resistance: Nanomedicines designed for immune stimulation can significantly enhance the activation of immune responses. This results in stronger and more specific immune reactions, making them effective in vaccines, cancer immunotherapies, and treatments for infectious diseases. Nanoparticles can help overcome immunotherapy resistance by addressing immunosuppressive signals within the tumor microenvironment, making it more responsive to immune attacks. Nanomedicines can be designed and applied to significantly enhance the immune response. This includes stronger T-cell activation, antibody production, and antigen presentation, thereby strengthening the immune system's ability to respond to disease.
4. Combination Therapies: The ability to deliver multiple agents in a single nanoparticle enhances the synergistic effects of combination therapies, improving overall treatment outcomes.
5. Long-Term Immune Memory: Some nanoparticles can promote long-lasting immune memory, potentially preventing tumor recurrence. By controlling the release of the drug, nanomedicines enable continuous immune stimulation and prolong the durability of the therapeutic effect. This is particularly important for the treatment of chronic diseases. Sustained antigen release and immunomodulatory properties of nanomedicines help create a more durable immune memory. This is crucial for vaccines and long-term protection against recurring infections or diseases like cancer.
6. Individualized therapy: The flexibility in the design of nanomedicines allows for individualized therapy based on patient-specific needs, including targeting specific immune cell subpopulations or the use of specific immunostimulants.
7. Improved Vaccine Efficacy: In the context of tumor vaccines, nanomedicines can improve vaccine efficacy by optimizing antigen presentation and immune response. They are particularly useful in developing new-generation vaccines against challenging diseases.

There is no doubt that tumor immunotherapy has made significant achievements in the clinic and has shown a promising future in cancer treatment. However, its widespread clinical application is significantly restrained because of the low efficacy against tumors and possible side effects. What’s important, the presence of immunosuppressive TME is the main reason for the low response rate, it is mostly attributed to the lack of tumor cells immunogenicity and tumor-infiltrating T lymphocytes.

Increasing studies have found that tumor inhibition microenvironment remodeling and cancer sensitization to immunotherapy can be achieved by promoting ICD induction at the tumor site. In the review, we firstly expound the definitions, molecular mechanisms and characteristics of ICD modalities in the first place, containing ferroptosis, pyroptosis and autophagy, and then summarize their crosstalk with antitumor immune responses as well as their application prospects in cancer therapy. Besides, we discuss potential mechanisms of nanoparticles-based ICD and outline current application of nanomaterials, nanoparticles and nano-drug delivery systems. These articles have revealed a more complex interrelation between immunogenic cell death and antitumor immune responses in various tumor therapies. Thus, it can be expected that nanomaterials-based technology modulates the modality and the immunogenicity of tumor cells death via the interaction between the utilization of nanoparticles and disruption with the removal of dying tumor cells.

Nevertheless, the impacts of ferroptosis, pyroptosis and autophagy on tumor immunotherapy are still uncertain as they enhance the antitumor immunity while antagonizing antitumor immunity. Furthermore, the interplays between cytokines and DAMPs released by ICD, as well as effectors such as RIPK1/3 and inflammasomes, and the immune system remain controversial. Therefore, it is critical to Figure out how the interactions among immune cells, cancer cells and stromal cells, three types of cells in the TME, influence tumor development [283]. What’s more, there are still several disadvantages in the utilization of nano-drug delivery systems, the top one is cytotoxicity deriving mainly from the size, material, concentration and surface charge of nanoparticles. Synthetic nanoparticles could be susceptible to phagocytosis and degradation by immune cells. Moreover, some nanoparticles that are difficult to be degraded could be stranded in the liver, lung, kidney, and other organs, resulting in irreversible damage. From the perspective of commercialization, the sophistication and difficulty of the synthesis of nanoparticles can further hinder their use in clinic [284, 285].

The most important obstacle to the application of nanotechnology and nanodrug delivery in cancer treatment is finding the most effective target able to trigger immune response. As a result, we need to make improvements in nanomaterials design and synthesis by mastering more profound understandings about the mechanisms of ICD. Thereby, researches of ICD and nanoparticles-based therapeutics should be carried out simultaneously, which mostly depends on the feasibility of new nanoparticles. In the future, it is required to exploit novel technologies capable of distinguishing different ICD modes. To be specific, it is necessary to Figure out the effect of each combination therapy on the immunosuppressive tumor microenvironment, which is important for the realization of outstanding therapeutic effect. Due to the limited number of stimuli that are able to induce ICD, it is essential to find more chemical compounds or cell death modalities that are able to effectively induce ICD. The development of new nanoparticles is also critically needed to achieve the tumor-targeted delivery of ICD inducers.

In conclusion, based on ICD induction, the implementations of nanoparticles are capable of achieving further advancement in the field of tumor immunotherapy, on account of their interdisciplinarity, interconnectivity and interdependence with immune-oncology.

## Data Availability

Not applicable.

## Abbreviations

AA: Arachidonic acid
ACSL: Acyl-CoA synthetase long-chain family member
ACT: Adoptive cell therapy
Acyl-CoA: Acetoacetyl coenzyme A
AMPK: 5′-AMP-activated protein kinase
ARG: Arginase
ATG: Autophagy-related gene
ATP: Adenosine-triphosphate
APCs: Antigen-presenting cells
BMDCs: Bone marrow-derived cells
CAR-T: Chimeric Antigen Receptor T-Cell Immunotherapy
CDK: Cyclin-dependent kinase
CDT: Chemodynamic therapy
Ce6: Chlorine e6
CEL: Celastrol
CQ: Chloroquine
CRT: Calreticulin
CTLA: Cytotoxic T-Lymphocyte Associated Antigen
CTLs: Cytotoxic T lymphocytes
CUR: Curcumin
DAMPs: Damage associated molecular patterns
DAC: Decitabine
DCs: Dendritic cells
DHA: Dihydroartemisinin
DNA: DeoxyriboNucleic Acid
DOX: Doxorubicin
ER: Endoplasmic reticulum
FAL: Pardaxin
Flav: Flavopiridol
FPN1: Ferroportin1
FTO: Fat mass and obesity-associated
GBM: Glioblastoma multiforme
GC: Gastric cancer
GOx: Glucose oxidase
GPX: Glutathione peroxidases
GSDMs: Gasdermins
GSH: Glutathione
HIP1R: Huntingtin-interacting protein 1-related
HMGB: High Mobility Group Protein
ICBs: Immune checkpoint blockers
ICD: Immunogenic cell death
ICG: Indocyanine green
IFN: Interferon
IL: Interleukin
IONP: Iron oxide nanoparticles
JAK: Janus kinase
LAP: LC3-associated phagocytosis
LPO: Lipid hydroperoxide
MA: Meclofenamic acid
mAb.: Monoclonal antibody
MDSCs: Myeloid-derived suppressor cells
MHC: Major histocompatibility complex
MIT: Mitoxantrone
MOF: Metal-organic framework
MRI: Magnetic resonance imaging
mTORc1: Target of Rapamycin complex 1
NBR1: Autophagy Cargo Receptor
NF-kB: Nuclear factor κB
NIR: Near infrared
NK: Natural killer
NLRP3: NOD-like receptor protein 3
NRF2: Nuclear Factor erythroid 2-Related Factor 2
NSCLC: Non-Small Cell Lung Cancer
OS: Osteosarcoma
OXA: Oxaliplatin
PD-1: Programmed death-1
PD-L1: Programmed cell death 1 ligand 1
PDT: Photodynamic therapy
PEG: Poly-ethylene glycol
PGE2: Prostaglandin E2
PI3P: Phosphatidylinositol 3-phosphate
PI3K/Akt/mTOR: Phosphoinositide 3-kinase/ protein kinase B/ Target of Rapamycin
PTI: Photoimmunotherapy
PLT: Platelet
PSN: P-selectin-binding peptide
PTAs: Photothermal conversion reagents
PTT: Photothermal therapy
PTX: Paclitaxel
PUFAs: Polyunsaturated fatty acids
Pyro-Fe: Pyrochlorophyll-Fe
RCD: Regulated cell death
RGX-104: Abequolixron
RIPK1: Receptor-interacting protein kinase 1
RNA: Ribonucleic acid
ROS: Reactive oxygen species
RSL-3: Glutathione peroxidases-4 inhibitors
SARs: Selective autophagic receptors
SAS: Sulfasalazine
SLC7A11: Solute carrier family 7, Member 11
SLC3A2: Solute carrier family 3, Member 2
SMAD: Drosophila mother against decapentaplegic protein
SOD2: Superoxide dismutase2
STAT1: Signal transducer and activator of transcription 1
TA: Tannic-acid
TAAs: Tumor-associated antigens
TAMs: Tumor-associated macrophage cells
TCR: T cell receptor
TGF-β1: Transforming growth factor-β
TIME: Tumor immune microenvironment
TLR4: Toll-like receptor 4
TME: Tumor microenvironment
TIR: Toll/IL-1 receptor
TIL: Tumor Infiltrating Lymphocytes
TRIF: TIR domain containing adaptor inducing interferon
Tregs: Regulatory cells
ULK: Unc-51-like kinase
VPS: Vacuolar protein sorting
